# Vulnerability of Breeding Waterbirds to Climate Change in the Prairie Pothole Region, U.S.A

**DOI:** 10.1371/journal.pone.0096747

**Published:** 2014-06-13

**Authors:** Valerie Steen, Susan K. Skagen, Barry R. Noon

**Affiliations:** 1 United States Geological Survey, Fort Collins Science Center, Fort Collins, Colorado, United States of America; 2 Department of Fish, Wildlife, and Conservation Biology, Colorado State University, Fort Collins, Colorado, United States of America; 3 Graduate Degree Program in Ecology, Colorado State University, Fort Collins, Colorado, United States of America; University of Regina, Canada

## Abstract

The Prairie Pothole Region (PPR) of the north-central U.S. and south-central Canada contains millions of small prairie wetlands that provide critical habitat to many migrating and breeding waterbirds. Due to their small size and the relatively dry climate of the region, these wetlands are considered at high risk for negative climate change effects as temperatures increase. To estimate the potential impacts of climate change on breeding waterbirds, we predicted current and future distributions of species common in the PPR using species distribution models (SDMs). We created regional-scale SDMs for the U.S. PPR using Breeding Bird Survey occurrence records for 1971–2011 and wetland, upland, and climate variables. For each species, we predicted current distribution based on climate records for 1981–2000 and projected future distributions to climate scenarios for 2040–2049. Species were projected to, on average, lose almost half their current habitat (-46%). However, individual species projections varied widely, from +8% (Upland Sandpiper) to -100% (Wilson's Snipe). Variable importance ranks indicated that land cover (wetland and upland) variables were generally more important than climate variables in predicting species distributions. However, climate variables were relatively more important during a drought period. Projected distributions of species responses to climate change contracted within current areas of distribution rather than shifting. Given the large variation in species-level impacts, we suggest that climate change mitigation efforts focus on species projected to be the most vulnerable by enacting targeted wetland management, easement acquisition, and restoration efforts.

## Introduction

The Prairie Pothole Region of north-central North America (central Iowa, U.S.A. to central Alberta, Canada; 900,000 km^2^) contains one of the largest wetland areas (40,000 km^2^) in the world [1]. Historically, most conservation activities have focused on sustaining extensive, high quality duck habitat because of the associated recreational value of duck-hunting across the U.S. [2]. Increasingly, emphasis is being placed on the diversity of ecosystem services offered by prairie pothole wetlands, including carbon sequestration, flood control, groundwater recharge, water quality improvement, and biodiversity [2]. This includes increasing attention to all 115 species of breeding or migrating waterbirds that depend on the region [3].

Successful management of species requires knowledge of habitat preferences. Strategic management of species also requires identifying those species most vulnerable to future threats. Land conversion continues to be a direct threat to waterbird habitat, but climate change will likely exacerbate loss and interact with changes in land cover. Climate models for the Prairie Pothole Region project increasing temperatures and slight or no increases in precipitation, indicating drier conditions affecting hydroperiods, and the extent and quality of wetland habitat [4], [5].

Prairie pothole wetlands are susceptible to climatic variation through impacts on wetland hydroperiod, vegetative condition, and water depth in combination with static factors such as basin size [5]. Well-documented causal relations between past variability in wetland condition and extent and waterbird numbers provide insights to future change in waterbird populations under climate change. In dry years, with fewer wet basins, breeding populations of waterbirds are significantly reduced [6], [7]. Building on these causal relations, Sorenson et al. [8] projected population changes for waterfowl under future warming scenarios. Their projections indicated that by 2060 duck populations would be half of their current level. Johnson et al. [5] used mechanistic models relating climate to marsh vegetation dynamics, and projected that the Prairie Potholes in North and South Dakota will be too dry to produce suitable wetland vegetative conditions for breeding ducks in the future.

To address how climate change may impact waterbirds in the Prairie Pothole Region, we created empirically-based species distribution models for a focal group of breeding wetland-associated birds. We related bird occurrence (presence/absence) to climate and land cover predictors. As a species' occurrence varies from year to year in response to dynamic wetland conditions, we used multiple years of bird survey data across 41 years, a period that included years of drought and years of heavy precipitation. Although we did not explicitly model wetland condition, we used Random Forests, an ensemble decision tree approach which can capture the interactions between climate variability and the state of wetland basins that drive wetland condition [9]. We projected future waterbird occurrence using species distribution models and future climate projections. To assess how climate change may reduce or expand current suitable habitat, for each species we compared the projections of future distribution to their predicted current distribution, and produced a quantitative estimate of how much habitat would be lost or gained under various climate change scenarios. Additionally, we compared our future projections of waterbird species response to a historic dry period.

## Methods

### Study Area

**Figure 1:**
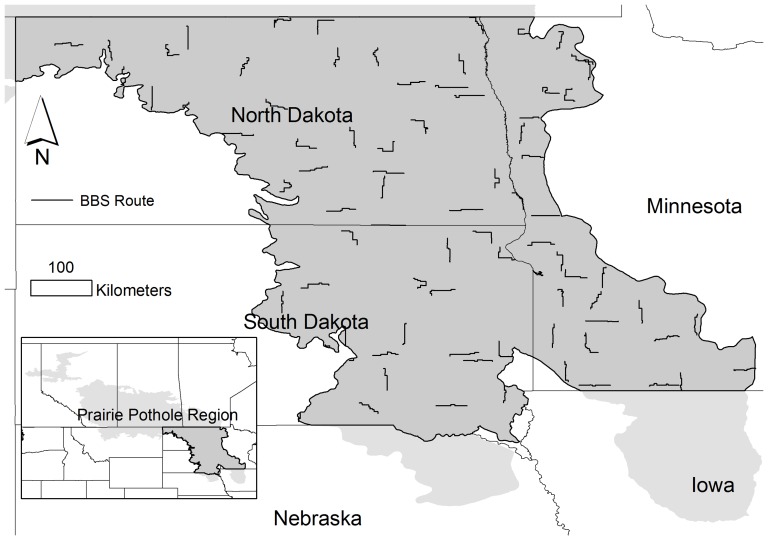
Bird occurrence data were obtained from 77 Breeding Bird Survey (BBS) routes throughout the Prairie Pothole Region (PPR) of North Dakota, South Dakota, and Minnesota. Climate-based projections were also made to the PPR of Iowa.

The study area (320,000 km^2^) was the 45% of the Prairie Pothole Region within four U.S. states (North Dakota, South Dakota, Minnesota, Iowa, Figure 1). The study was restricted to the four states because of available and consistent land cover and downscaled climate data. We excluded Iowa from training the model because too few wetlands remain there to usefully inform the species distribution model, although we did include it in model predictions.

Water-filled glacial depressions termed *potholes* are characteristic of this region and can reach densities greater than 40 per km^2^ [10]. Since European settlement, these wetlands have been extensively converted to cropland, with wetland losses greatest in the eastern portion of the Prairie Pothole Region: Minnesota (85%), Iowa (95%) and North (49%) and South Dakota (35%) [11], [12]. Losses of surrounding upland prairie habitats follow a similar geographic gradient (greatest in the eastern portion of the Prairie Pothole Region) but have been even more severe than wetland losses [3].

### Species Occurrence

We obtained species occurrence (presence/absence) data from the North American Breeding Bird Survey (BBS) [13] for waterbirds species with a prevalence of ≥ 0.05. The BBS consists of >3000 routes on secondary roads throughout the continental U.S. and southern Canada. Routes are surveyed once annually during June between 04.45 AM and 10.00 AM. Route locations generally remain the same year after year, although not all routes are surveyed each year and there is variation in the year when a route is initiated. BBS routes are 39.4-km long with 50 stops spaced 0.8 km apart. Three-minute point-count surveys are conducted at each stop. BBS survey data are available for each species and summarized at route totals or 10-stop route segments (https://www.pwrc.usgs.gov/bbs/).

In our study area, BBS surveys took place from late May to early July. This interval extensively overlapped the breeding season (nest-building through brood rearing) for the majority of wetland-dependent species we evaluated. Ten species usually nest during this period and three species occupy brood-rearing habitats. The remainder of the species are engaged in behaviors ranging from incubation to brood-rearing. In addition, seven waterfowl species may be molting body or primary feathers near the end of the survey period.

Even though the breeding cycles of wetland-dependent birds in the Prairie Potholes are not completely synchronous, we believe the BBS survey methods accurately document the presence of all regularly occurring species. Our confidence is based on the overlap between the geographic extent of our survey data, the distribution of our focal species during the breeding season, and the timing of the surveys. The result, we believe, is that the likelihood of correctly documenting the presence of a species was comparable across species, routes, and survey years.

We used data (1971–2011) from high-quality surveys (reported by the BBS as “run type 1”) for 77 routes: these were conducted within the correct survey window and not during poor weather. Due to the potential for extensive variation along a route in habitat types, we chose one 10-stop section to model habitat associations rather than use data from the entire route. To accommodate different timing of peak detectability by species, we chose either the first or third section for a species depending which section had higher detections for that species. Routes were consistently surveyed from stop one, starting around 04.45 AM, to stop 50, ending around 09.00 AM. For all but two species, the first or third section had their highest or second highest number of detections. ‘Presence’ was defined as ≥1 detection at a minimum of one stop along the route segment. We identified focal species based on their prevalence (section-level occurrence rate) with species detected at fewer than 5% of route sections not included.

### Land Cover Data

We extracted land cover variables (Table 1) for North and South Dakota from a GIS raster layer created by the U.S. Fish and Wildlife Service (USFWS; USFWS, Region 6 Habitat and Population Evaluation Team, unpublished data); for Minnesota and Iowa from a GIS raster layer created by the USFWS (USFWS, Region 3 Habitat and Population Evaluation Team, unpublished data); and for uplands in the southern portion of the Iowa Prairie Pothole Region from the 1992 National Land Cover Dataset (NLCD). The USFWS data layers were based on Landsat Thematic Mapper Satellite imagery of scenes from 2000–2003, and the NLCD on scenes from the early to mid-1990s. All raster layers were at a 30-m resolution.

**Table 1:** Thirty-one climate and land cover variables used in species distribution models.

Climate		Land Cover	
Temperature	Precipitation	Wetland	Upland
Spring (spr)	Spring	Temporary (temp)	Cropland (crop)
Winter (wint)	Winter	Seasonal (seas)	Grassland (grass)
Fall	Fall	Semipermanent (semi)	Developed (devel)
Summer (sum)	Summer	Lake	Tree
Yearly (1yr)	Yearly	River	
5-year (5yr)	5-year	Shrub	
10-year (10yr)	10-year	Forested (forest)	
5-year std. dev. (5yr_sd)	5-year std. dev.	Total palustrine (pal)	
10-year std. dev. (10yr_sd)	10-year std. dev.	Total	

Temperature calculations were based on averages, while precipitation calculations were based on totals. Land cover variables were based on composition (proportion of total) of that cover type in the landscape. Wetland land cover was apportioned by wetland regime. Total palustrine wetland summed temporary, seasonal, and semipermanent wetlands. Total wetland summed all wetland regimes. Cropland described land planted with crops or fallowed. Grassland included native prairie, conservation reserve program (CRP) land, and hayland.

Wetland basins in the land cover layers were areas of contiguous wetland extent. The basins were derived from a GIS wetland polygons layer (USFWS National Wetlands Inventory, NWI) where multiple contiguous polygons of differing wetland regimes were dissolved to a single polygon. The USFWS Habitat and Population Evaluation Team followed the procedures of Cowardin et al. [14] and Johnson and Higgins [15] to describe each wetland basin by its most permanent water regime: temporary, seasonal, semipermanent, lake, and river. Generally, temporary wetlands are flooded in spring for a few weeks after snow-melt, seasonal wetlands hold water until summer, and semipermanent wetlands hold water through the growing season; lake and rivers are permanently flooded wetlands [16]. NWI data are based on aerial photographs taken in the late 1970’s and early 1980’s. Where water pixels extended beyond NWI polygons, they were labeled as water (wetland regimes, see Cowardin et al. [17]). We characterized wetlands into nine classes: temporary, seasonal, semipermanent, lake, river, forested, shrub, total, and total palustrine (Table 1). Total wetland was the combined composition of temporary, seasonal, semipermanent, lake, river, forested, and shrub; total palustrine wetland was temporary, seasonal, and semipermanent.

We described upland habitat using four land cover classes: cropland, grassland, tree, and developed (Table 1). Cropland included areas planted with crops or fallowed. Grassland included native prairie, planted grasses (i.e. previously cropped but now planted with grasses and forbs such as Conservation Reserve Program land), and hayland. Developed land cover included primarily residential areas. Tree habitat included small sections or rows of trees and occasionally areas of forest. Accuracy of the upland land cover data for North and South Dakota, assessed in 2007, was > 90% (M. Estey, personal communication).

To describe habitat associations for our focal waterbird species, we explored composition-based single scale models. In both single-scale and multi-scale models, composition-based predictors, expressed as the amount of a land cover type within a given area, perform better than their distance-based counterparts, expressed as the distance from a sampling location to a land cover type [18]. We used ArcMap 10.0 to calculate land cover composition for the four upland and nine wetland classes at six spatial scales for the BBS route segments. The scales ranged from 335 ha to 32,200 ha and were based on buffering the segments with radii: 0.2-km, 0.4-km, 1-km, 2-km, 4-km, and 8-km. BBS surveyors record all birds detected within 0.4-km of the survey point. Thus, assuming no decline in detection probability with increasing distance and no landscape effect, we expected 0.4-km to be the appropriate scale to relate land cover to bird occurrence. However, some waterbird species may decline quickly in detection probability with increasing distance from the survey point—therefore, we also explored a 0.2-km scale. Because other species may respond to land cover heterogeneity at broader extents, we also explored a range (1-km to 8-km) of landscape scales. Land cover data were assumed static across current and future years.

### Climate Covariates

We used PRISM (PRISM, Parameter-elevation Regressions on Independent Slopes Model) data for historical climate records. These data are available at a 4-km grid scale as monthly temperature and precipitation and were rescaled to an 8-km grid to match the scale of the projected climate data [19].

Using monthly values of precipitation and temperature, we derived 18 climate variables (Table 1). We calculated mean temperatures for grid points by averaging the minimum and maximum monthly temperatures over different time periods. We delineated seasons as summer (June-August), fall (September-November), winter (December-February), and spring (March-May). We defined year as ending in May to correspond to the June bird surveys. We included seasonal and annual variables because both seasonal and annual climate explain annual variation in the number of prairie pothole wetlands holding water [20]. For semipermanent wetlands and (especially) lakes, wet wetland count is related to long-term climate (at least 3 years) [21]. We included 5-year and 10-year precipitation and mean temperature variables as proxies for long-term climate effects. We also included the variances in 5-year and 10-year precipitation and temperature data, because large values of these variables may indicate that wetlands are cycling through wet and dry phases, driving dynamic vegetative conditions [5]. Climate data from 1971–2011 were used to construct the baseline species distribution models. The species distribution models predicted to climate data from 1981–2000 and 2040–2049 to create current and future projections, respectively, of species occurrence.

### Future Climate Data

We used statistically downscaled and high resolution climate projections. Statistically downscaled data refine projections from global circulation models (GCMs) using an empirical relationship to local physiography (e.g. topography and water bodies). These projections assume relations will hold into the future and are less computationally intensive than high resolution models. High resolution models nest a dynamical Regional Climate Model within the GCM, re-running the GCM based on mesoscale (a few to a few hundred kilometers) physical relationships with topographical features and surface characteristics [22]. The high resolution projections circumvent the problem associated with lack of “stationarity” when the relationships between GCM output and the fine-scale climate change over time.

The statistically downscaled projections were based on data obtained from output of GCM CGCM3.1MR (Canadian Centre for Climate Modeling and Analysis Third Generation Coupled Global Climate Model Version 3.1, Medium Resolution) [23] and downscaled to an 8-km grid. The high resolution models used the Community Climate System Model (CCSM) to set the boundary condition and a mesoscale model, Weather Research and Forecasting model (WRF) to refine the data to a 36-km regional scale (J. Stamm, personal communication) [24]. Given that we expected high spatial correlation for monthly temperature and precipitation, we interpolated the 36-km data to the 8-km grid [25]. Both climate models were run with a mid-high IPCC emissions scenario, A2 [26]. The high resolution projections were available for 2000–2049, and the statistically downscaled projections were available for 2000–2100. We term the statistically downscaled data “CGCM” after the GCM these data are based on, and we term the dynamically downscaled data “WRFc” after the mesoscale model these data are based on.

### Species Distribution Models

We estimated a species distribution model (SDM) for each waterbird species, relating BBS occurrence records (1971–2011) to climate, and wetland and upland land cover (hereafter grouped as “land cover”) predictor variables. We used climate for the same year as the occurrence record from the climate grid point nearest the BBS route segment and land cover surrounding the route segment. We defined occurrence as one or more detection per 10-stop segment by year. The spatial scale used in the final models for land cover calculations was chosen separately for each species based on model performance. We ran six models for each species based on the six different spatial scales of land cover and chose the model with the highest classification accuracy. We used a non-parametric machine learning approach, Random Forests, to create the SDMs [9]. We chose Random Forests because of its high predictive power, ability to model unspecified variable interactions and correlated variables, its ranking of variable importance, and its demonstrated use for bioclimatic species distribution models [27], [28]. Random Forests uses an ensemble of classification (categorical response variable) or regression (continuous response variable) trees, each built with a subset of the data, to model the pattern between predictor variables and the response variable. We used permutation procedures to assess variable importance, a method based on reduction in predictive accuracy to internally withheld data when values of a given predictor variable are randomly shuffled. We report the top ten variables for each model. Although the choice of the number of variables to report is arbitrary, we expect the top ten will provide an adequate basis for comparing models.

We used the RandomForests package in R to create our models [29]. We specified 3000 trees which is a sufficiently large number of trees to capture any patterns in the data. Each tree was constructed with a bootstrapped subsample with replacement of the data records (BBS routes). Because the ratio of presence to absence was often skewed, particularly for either very abundant or rare species, we balanced the data by setting Random Forests to randomly use, for each tree, 25 records where the species was present and another 25 where the species was absent [30]. A subsample of five predictor variables was evaluated at each binary split in the tree algorithm.

We partitioned the BBS data in a number of ways to strengthen model evaluation and inference. First, we only excluded consecutive years of surveys to reduce the influence of temporal autocorrelation and maximize information content: the “main training set”. The excluded data were used to validate the models created with these data: the “main test set”. Second, we separated out six years of data covering a drought period from 1987 through 1992 [21]. We created species distribution models with the drought data to look at variable importance in dry years compared to variable importance for the whole study period (main training set). To assess model transferability, we predicted to the drought data subset using species distribution models created with the remaining wetter years [31], [32].

### Model Evaluation

To evaluate each model's ability to forecast to the same range of predictor variables, we predicted to the main test set. To evaluate each model's transferability – that is, to project to a new location or time period where predictor variables may be outside the range of the variables used to build the model – we projected to the drought period with models trained with data from the wet years. The transferability assessment should more realistically evaluate how the models extrapolate to a dry future [31].

To assess a model's performance, we report patterns of correct classification in a confusion matrix and the area under the receiver operating characteristic curve (AUC) [33]. From the confusion matrix, we report the counts of true positives, false positives, true negatives, false negatives and overall classification accuracy based on a 0.5 probability of occurrence threshold for concluding presence. Because we set sample sizes of presence and absence points to be equally subsampled in the Random Forests model, we selected a threshold of 0.5 [34]. Overall classification accuracy was calculated by dividing the number of correctly predicted presences and absences by total predictions. AUC is a threshold free assessment of model performance. AUC values range from zero to one and give the probability that a known presence observation has a higher predicted value of presence than an absence observation for a randomly selected pair of presence-absence observations [33]. Models with AUC values of at least 0.7 are considered acceptable, between 0.8 and 0.9 good, and greater than 0.9 outstanding [35].

### Projected Distributional Changes

We created current predictions and future projections of probability of occurrence to each grid cell, for each focal species, by applying the SDMs to the baseline land cover and climate data (the 20-year period for baseline climate data being 1981–2000) and to baseline land cover and future climate data (the 10-year period 2040–2049). Ten to twenty-year time periods were chosen to mitigate the influence of short-term variations in climate.

We created current and future predictive distribution maps for each species in ArcMap 10 based on an assignment of grid point locations as suitable or unsuitable. A grid point was determined suitable if the estimated probability of occurrence (over the time period for the baseline or future data) was greater than 0.5. Three breakpoints within suitable (0.625, 0.75, 0.875) and unsuitable (0.125, 0.25, 0.375) locations showed the degree to which a location was predicted suitable or unsuitable.

We indexed changes between predicted baselines and projected future distributions using change in a species' spatial distribution. To assess change in distribution, we calculated the percent loss (or gain) in the number of grid cells classified as suitable.

## Results

**Figure 2:**
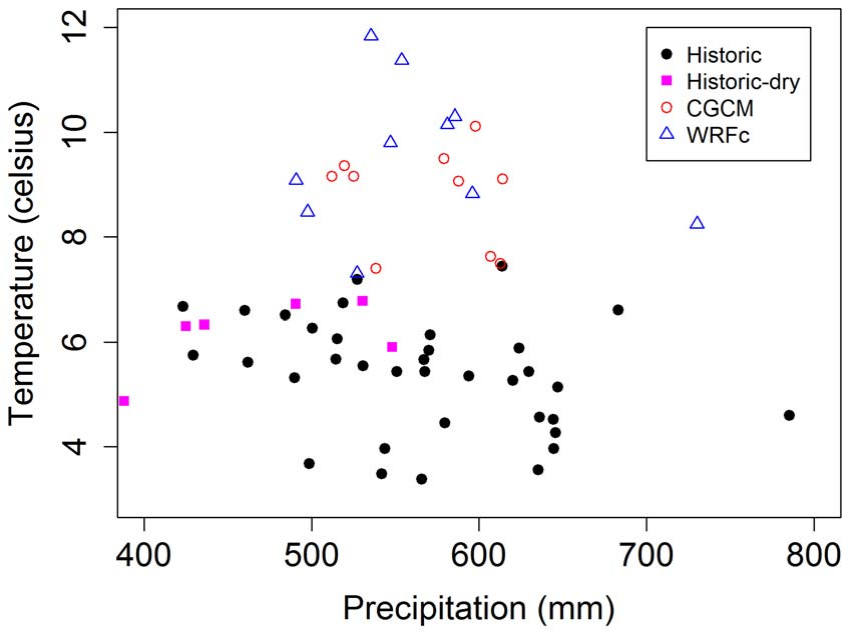
Total precipitation versus average temperature by “bird year” (June of year x-1 to May of year x) for the study area (see Figure 1) for the time periods used to train species distribution models and project future distributions. Historic points showed the years and locations from 1971–2011 used to train the species distribution models with six years withheld. The six years were a dry period from 1987–1992 shown as ‘historic-dry’. CGCM and WRFc show two sets of climate projections for the ten year period 2040-49.

**Figure 3:**
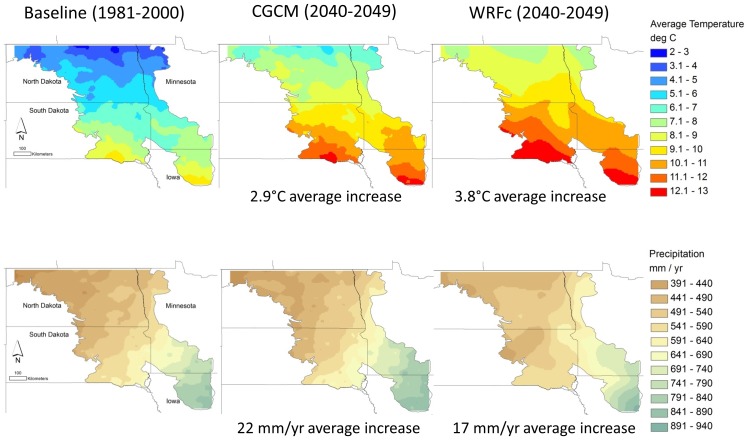
Temperature and precipitation for baseline and two future climate projections for the prairie potholes of North Dakota, South Dakota, Minnesota, and Iowa.

Baseline mean temperature (years 1981–2000) was 5.9°C and mean yearly precipitation was 548 mm. By 2040–2049, CGCM projected a 2.9°C increase in mean temperature and a 22 mm (3.9%) increase in annual precipitation while WRFc projected a 3.8°C temperature increase and a 17 mm (3.1%) increase in annual precipitation. Projections of future precipitation fall within the range of historic levels of precipitation, whereas future temperatures projected by both climate models exceed historic temperatures (Figure 2). The climate models differed slightly in the spatial distribution of the precipitation increases, with CGCM projecting greater increase in Iowa and WRFc projecting greater increase in North Dakota than other areas (Figure 3).

The number of data occurrence records in the main training set was 975. The number of years of survey data included in the main training set, for a given route, ranged from one to 21, with a mean of 13. The number of survey routes included for a given year ranged from 15 to 35. Thirty-one waterbird species had prevalence ≥ 0.05 and were included in the focal group (Table 2). The number of records in the main test set was 817.The number of data records in the dry years set was 139. We adjusted the prevalence cutoff to 0.07 (≥ 10 detections), at which 22 species qualified.

**Table 2:** Model evaluation showing species prevalence (proportion of data points with species present) for each dataset, and predictive accuracy using the classification matrix and area under the curve (AUC) values.

		Prevalence			To withheld data						To withheld dry years	
Common Name	Scientific Name	Training data (n = 974)	Withheld data (n = 817)	Dry years data (n = 139)	True positive (n)	False positive (n)	True negative (n)	False negative (n)	Overall accuracy (%)	AUC	Overall accuracy (%)	AUC
Canada Goose	Branta canadensis	0.18	0.18	0.09	92	211	457	60	67	0.71	76	0.61
Wood Duck	Aix sponsa	0.06	0.07	0.04	24	187	576	30	73	0.70	84	0.81
Gadwall	Anas strepera	0.11	0.12	0.00	82	183	534	18	75	0.89	74	0.87
American Wigeon	Anas americana	0.05	0.07	0.04	36	129	632	20	82	0.84	71	0.66
Mallard	Anas platyrhynchos	0.60	0.59	0.55	343	101	231	142	70	0.77	57	0.65
Blue-winged Teal	Anas discors	0.36	0.38	0.24	259	139	368	51	77	0.86	63	0.74
Northern Shoveler	Anas clypeata	0.17	0.18	0.09	117	189	481	30	73	0.84	68	0.81
Northern Pintail	Anas acuta	0.21	0.24	0.10	153	161	463	40	75	0.85	69	0.85
Green-winged Teal	Anas crecca	0.05	0.05	0.03	29	180	592	19	77	0.85	72	0.58
Redhead	Aythya americana	0.15	0.12	0.03	87	126	593	11	83	0.91	81	0.95
Ruddy Duck	Oxyura jamaicensis	0.13	0.11	0.06	83	115	612	7	85	0.94	83	0.92
Pied-billed Grebe	Podilymbus podiceps	0.22	0.24	0.11	166	150	474	27	78	0.90	73	0.81
Double-crest. Cormorant	Phalacrocorax auritus	0.09	0.09	0.05	64	201	542	10	74	0.84	68	0.75
American Bittern	Botaurus lentiginosus	0.24	0.27	0.17	190	173	421	33	75	0.84	64	0.76
Great Blue Heron	Ardea herodias	0.07	0.06	0.04	29	180	592	19	76	0.69	88	0.67
Sora	Porzana carolina	0.28	0.27	0.16	178	154	443	42	76	0.86	63	0.71
American Coot	Fulica americana	0.28	0.29	0.15	201	117	467	32	82	0.90	72	0.86
Killdeer	Charadrius vociferus	0.88	0.92	0.86	507	27	41	242	67	0.69	69	0.59
Upland Sandpiper	Bartramia longicauda	0.49	0.48	0.53	308	128	300	81	74	0.82	81	0.84
Willet	Tringa semipalmata	0.16	0.15	0.13	111	163	532	11	79	0.91	70	0.90
Marbled Godwit	Limosa fedoa	0.19	0.18	0.24	121	166	505	28	76	0.88	75	0.90
Wilson's Snipe	Gallinago delicata	0.19	0.20	0.14	140	163	494	23	77	0.90	72	0.80
Wilson's Phalarope	Phalaropus tricolor	0.10	0.12	0.04	79	165	550	23	77	0.86	66	0.84
Franklin's Gull	Leucophaeus pipixcan	0.10	0.11	0.09	64	189	538	26	74	0.81	73	0.86
Ring-billed Gull	Larus delawarensis	0.12	0.16	0.12	103	179	510	25	75	0.84	66	0.78
Black Tern	Chlidonias niger	0.17	0.17	0.07	112	172	505	28	76	0.85	72	0.89
Sedge Wren	Cistothorus platensis	0.27	0.26	0.17	142	168	434	73	71	0.76	81	0.71
Marsh Wren	Cistothorus palustris	0.23	0.24	0.17	166	130	493	28	81	0.89	73	0.78
Common Yellowthroat	Geothlypis trichas	0.83	0.84	0.81	444	41	91	241	65	0.74	60	0.76
Song Sparrow	Melospiza melodia	0.66	0.66	0.57	425	105	172	115	73	0.78	74	0.85
Yellow-headed Blackbird	Xanthocephalus xanthocephalus	0.54	0.56	0.56	348	86	321	62	82	0.88	64	0.76

The positive and negative rates show the models ability to correctly predict presence and absence data points in the withheld data based on a 0.5 threshold for classification. Overall accuracy was the proportion of true positive and true negative predictions. AUC critical value  =  0.70. Dry year predictions were based on models trained without the dry years.

### Model Evaluation

Most models based on known distributional patterns were acceptable to excellent, indicated by AUC values (Table 2). Exceptions were SDMs for the Great Blue Heron and Killdeer. When predicting to dry years only, AUC values indicated the following additional models predicted poorly: Canada Goose, American Wigeon, Mallard, and Green-winged Teal. Overall accuracy of dry year predictions suggested that projected distributional changes for some species should be interpreted with caution, including Blue-winged Teal, Sora, and Common Yellowthroat. For the main datasets, model performance was not related to a species' prevalence according to Spearman's rank correlation (-0.09, p-value 0.62).

### Vulnerability

Average projected decline in occurrence rate (spatial distribution) across 31 species under two future climate scenarios was 45%. WRFc models projected slightly more severe distributional changes (-48%) than CGCM (-43%; Table 3). Species expected to experience small to no declines in distribution included Blue-winged Teal, Killdeer, and Upland Sandpiper. Species projected to experience severe declines were Franklin's Gull, Sora, and Wilson's Snipe (Table 3). In general, species maps depicted declines within the baseline range, rather than distributional shifts to new areas (Figures S2-S12).

**Table 3:** Values report projected changes in occurrence in the 2040’s, relative to 1981–2000 (baseline).

Species	Change in occurrence (%)		
	CGCM	WRFc	Average
**Canada Goose**	**−76**	**−66**	**−71**
**Wood Duck**	**−70**	**−37**	**−54**
**Gadwall**	**49**	**−87**	**−19**
**American Wigeon**	**−58**	**−71**	**−65**
**Mallard**	**−30**	**−23**	**−27**
**Blue-winged Teal**	**−9**	**−4**	**−7**
**Northern Shoveler**	**−51**	**−62**	**−57**
**Northern Pintail**	**−45**	**−37**	**−41**
**Green-winged Teal**	**−46**	**−18**	**−32**
**Redhead**	**−42**	**−35**	**−39**
**Ruddy Duck**	**−30**	**−31**	**−31**
**Pied-billed Grebe**	**−40**	**−18**	**−29**
**Double-crested Cormorant**	**−11**	**−20**	**−16**
**American Bittern**	**−42**	**−42**	**−42**
**Great-blue Heron**	**−72**	**−82**	**−77**
**Sora**	**−94**	**−98**	**−96**
**American Coot**	**−38**	**−38**	**−38**
**Killdeer**	**5**	**0**	**3**
**Upland Sandpiper**	**8**	**7**	**8**
**Willet**	**−43**	**−58**	**−51**
**Marbled Godwit**	**−53**	**−61**	**−57**
**Wilson's Snipe**	**−99**	**−100**	**−100**
**Wilson's Phalarope**	**−42**	**−60**	**−51**
**Franklin's Gull**	**−93**	**−98**	**−96**
**Ring-billed Gull**	**−39**	**−83**	**−61**
**Black Tern**	**−67**	**−64**	**−66**
**Sedge Wren**	**−71**	**−60**	**−66**
**Marsh Wren**	**−40**	**−42**	**−41**
**Common Yellowthroat**	**−26**	**−35**	**−31**
**Song Sparrow**	**−38**	**−41**	**−40**
**Yellow-headed Blackbird**	**−24**	**−25**	**−25**

Species distribution models projected species occurrence to 4,957 8-km grid points using climate data for the baseline period and two climate projections (CGCM and WRFc). Negative values indicated the proportion of occupied grid cells for each species, projected to be unoccupied in the future. Positive values indicated the proportion by which occupied cells were projected to increase.

**Figure 4:**
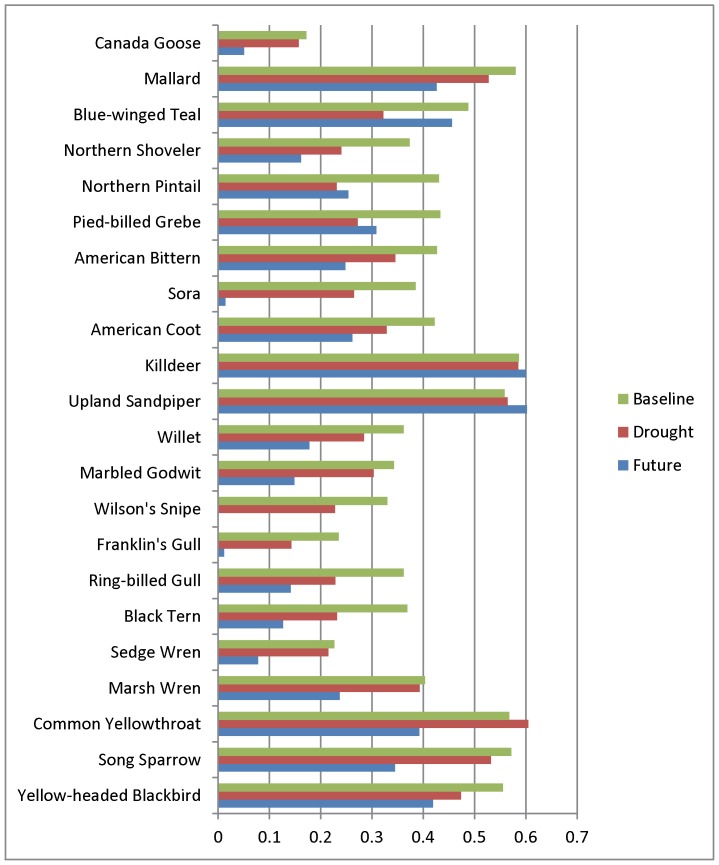
Mean rates of predicted species occurrence at 4,957 8-km grid points. Baseline rate was based on 1981–2000 climate records. Dry years showed predicted occurrence rates for the drought period, 1987–1992. Future rates were based on the average projections of two future climate datasets (CGCM-A2 and WRFc) for 2040–2049.

For most species, future projections of change were consistent with responses of species to historic dry periods (Figure 4). Consistent projections were those that exhibited little to no change between the historic dry period and the future, or those that declined more in the future than in the historic dry period. If a species' habitat was not impacted by dry conditions, the species would be expected to experience little to no impact under future dry conditions. Other species may be impacted by drying conditions, thus responding during the historic dry period, and even more if additional drying occurs in the future. However, inconsistent with expectations, models projected reduced distribution of Blue-winged Teal, Northern Pintail, and Pied-billed Grebe in the dry period relative to future projections. Additionally, several species that remained relatively stable in the historic dry period were projected to decrease in distribution under future scenarios, including Canada Goose, Sedge Wren, Marsh Wren, Common Yellowthroat, and Song Sparrow.

### Variable Importance

In general, species distributions were strongly influenced by the distribution of wetland basins and land cover classes and moderately influenced by climate, as evidenced by their influence in the SDMs. Land cover variables, wetland and upland, collectively occurred as 67% of the top ten variables in the SDMs but comprised only 42% of the available predictor variables (Table 4); wetland and upland variables were 1.5 and 1.8 times more likely to appear in lists of top ten predictors than in the list of available predictors, respectively. Species associations with all wetland types, except rivers, were generally positive. All associations with cropland were negative except for the Song Sparrow, whereas associations with grassland were primarily positive except for Wood Duck (Table 4). Climate predictor variables were generally underrepresented in the variables of top importance. Collectively, temperature and precipitation comprised 32% of the top ten variables across the 31 species, although they were 58% of the available predictor variables. Temperature and precipitation variables were similarly influential and were 0.5 and 0.6 times more likely to appear in lists of top ten predictors than in the list of available predictors, respectively. In general, probability of species occurrence was negatively associated with temperature; relationships with temperature variability were often positive (Table 4). Associations with precipitation were often negative, except for Great Blue Heron, Sedge Wren, Song Sparrow, and Wood Duck. Variability in precipitation occurred in the top ten variables for only one species' model (Sedge Wren) and was negatively correlated with probability of occurrence.

**Table 4:** Variable importance for Random Forest species distribution models.

Rank	Canada Goose	Wood Duck	Gadwall	American Wigeon	Mallard	Blue-winged Teal	Northern Shoveler	Northern Pintail	Green-winged Teal	Redhead	Ruddy Duck
1	W-lake(+)	W-temp(-)	T-5yr_sd(-)	W-lake(+)	W-pal(+)	W-total(+)	W-pal(+)	U-tree(-)	U-tree(-)	W-semi(+)	W-semi(+)
2	P-wint(+)	P-wint(+)	P-10yr_sd(-)	U-tree(-)	W-semi(+)	W-pal(+)	W-total(+)	W-pal(+)	P-wint(+)	W-total(+)	W-total(+)
3	W-river(-)	U-grass(-)	U-tree(-)	W-semi(+)	W-river(-)	W-seas(+)	W-semi(+)	P-10yr(-)	W-lake(+)	W-lake(+)	W-lake(+)
4	T-sum(-)	U-crop(-)	T-spr(-)	W-temp(-)	W-total(+)	W-semi(+)	W-temp(+)	W-seas(+)	W-seas(+)	U-tree(-)	W-pal(+)
5	T-spr(-)	U-tree(+)	U-grass(+)	P-5yr(-)	T-10yr_sd(+)	U-grass(+)	W-seas(+)	W-semi(+)	U-crop(-)	W-temp(-)	U-tree(-)
6	T-10yr_sd(+)	U-devel(-)	P-10yr(m)	P-spr(-)	W-temp(+)	W-temp(+)	U-tree(-)	P-5yr(-)	P-fall(∼)	W-pal(+)	W-seas(+)
7	T-10yr(m)	W-semi(+)	U-crop(-)	W-pal(+)	U-tree(-)	U-tree(-)	U-crop(-)	U-grass(+)	W-shrub(-)	W-river(-)	W-river(-)
8	T-5yr(m)	T-10yr_sd(+)	T-10yr_sd(+)	W-total(+)	T-spr(-)	W-river(-)	U-grass(+)	W-river(-)	W-temp(+)	W-seas(+)	W-temp(-)
9	T-1yr(-)	T-sum(∼)	W-total(+)	P-10yr(-)	U-grass(+)	U-crop(-)	T-spr(-)	T-spr(-)	P-5yr(-)	U-crop(-)	U-crop(-)
10	W-total(+)	W-total(+)	W-river(-)	P-1yr(-)	T-5yr_sd(+)	P-10yr(-)	P-10yr(-)	W-total(+)	T-5yr(-)	U-grass(+)	U-grass(+)
Rank	Pied-billed Grebe	Double-crested Cormorant	American Bittern	Great-blue Heron	Sora	American Coot	Killdeer	Upland Sandpiper	Willet	Marbled Godwit	
1	W-semi(+)	W-semi(+)	U-crop(-)	T-spring(-)	P-10yr(-)	W-semi(+)	W-semi(+)	W-seas(+)	U-tree(-)	U-tree(-)	
2	W-total(+)	U-tree(∼)	W-total(+)	P-10yr(+)	T-sum(-)	W-total(+)	W-pal(+)	U-grass(+)	P-10yr(-)	U-crop(-)	
3	W-pal(+)	W-temp(-)	U-grass(+)	U-tree(+)	T-5yr(-)	W-pal(+)	W-river(-)	U-tree(-)	P-5yr(-)	W-lake(+)	
4	W-seas(+)	T-10yr_sd(+)	W-semi(+)	P-5yr(+)	W-seas(+)	W-seas(+)	U-tree(-)	P-10yr(-)	U-grass(+)	W-total(+)	
5	U-crop(-)	W-lake(+)	W-pal(+)	P-spr(+)	T-1yr(-)	W-river(-)	W-total(+)	W-pal(+)	W-pal(+)	P-10yr(-)	
6	W-lake(+)	T-5yr(∼)	W-seas(+)	P-1yr(+)	W-river(-)	P-10yr(-)	W-seas(+)	U-devel(-)	W-semi(+)	U-grass(+)	
7	T-10yr_sd(+)	P-5yr(+)	P-10yr(-)	T-10yr(-)	W-total(+)	W-lake(+)	U-devel(-)	W-river(-)	W-total(+)	T-10yr(-)	
8	U-grass(+)	P-spr(-)	T-5yr(-)	W-shrub(+)	W-pal(+)	U-crop(-)	P-wint(∼)	U-crop(-)	U-crop(-)	P-5yr(-)	
9	T-sum(-)	P-fall(∼)	T-sum(-)	P-sum(+)	P-5yr(-)	U-grass(+)	W-temp(+)	W-semi(+)	T-10yr(-)	W-semi(+)	
10	U-tree(-)	T-5yr_sd(-)	T-1yr(-)	T-1yr(-)	U-grass(+)	T-5yr(-)	U-grass(+)	P-5yr(-)	W-river(-)	W-river(-)	
Rank	Wilson's Snipe	Wilson's Phalarope	Franklin's Gull	Ring-billed Gull	Black Tern	Sedge Wren	Marsh Wren	Common Yellow-throat	Song Sparrow	Yellow-headed Blackbird	
1	T-5yr(-)	U-tree(-)	U-tree(+)	W-semi(+)	W-seas(+)	P-10yr_sd(-)	W-semi(+)	W-total(+)	U-grass(-)	W-semi(+)	
2	T-10yr(-)	U-grass(+)	W-pal(+)	W-lake(+)	W-pal(+)	U-tree(+)	W-total(+)	W-temp(+)	U-crop(+)	W-pal(+)	
3	W-total(+)	U-crop(-)	T-10yr(-)	W-pal(+)	W-total(+)	U-crop(-)	W-pal(+)	W-seas(+)	U-tree(+)	W-total(+)	
4	U-crop(-)	P-10yr(-)	W-total(+)	W-total(+)	W-semi(+)	P-10yr(+)	U-crop(-)	W-pal(+)	P-10yr(+)	W-seas(+)	
5	W-pal(+)	P-5yr(-)	W-seas(m)	U-tree(-)	W-temp(+)	T-sum(-)	W-seas(+)	U-tree(+)	W-semi(-)	U-grass(+)	
6	W-lake(+)	W-lake(m)	U-grass(∼)	U-grass(+)	U-crop(-)	P-5yr(+)	U-grass(+)	W-semi(+)	P-sum(+)	P-10yr(-)	
7	T-sum(-)	W-semi(+)	W-temp(+)	P-5yr(-)	P-10yr(-)	T-10yr_sd(+)	W-lake(+)	U-devel(-)	T-10yr_sd(+)	U-crop(-)	
8	U-tree(∼)	W-seas(+)	W-lake(+)	W-seas(+)	T-5yr(-)	W-pal(+)	T-sum(-)	W-lake(+)	T-sum(-)	T-sum(-)	
9	W-river(-)	P-spr(∼)	U-crop(-)	W-river(-)	P-wint(+)	U-grass(+)	W-river(-)	T-5yr(-)	T-10yr(-)	U-tree(-)	
10	W-seas(∼)	W-total(+)	P-sum(∼)	T-5yr(-)	P-5yr(m)	P-1yr(+)	T-10yr(-)	P-5yr_sd(-)	P-5yr(+)	P-5yr(-)	

Top ten variables are shown in descending order of rank. Variable categories were denoted by W (wetland), U (upland), P (precipitation), and T (temperature). Signs indicated the relationship between the predictor and the species response: + (positive), - (negative), m (unimodel), and ∼ (equivocal).

Land cover variables were highly influential in observed patterns of species distribution. The importance of these variables is visually apparent when spatial distribution of grasslands and wetlands (Figure S1) and observed climate gradients (Figure 3) were compared to baseline distributions (Figures S2-S12). Many breeding waterbirds have a high probability of occurrence in the western portion of the study area where grasslands and wetlands co-occur.

**Figure 5:**
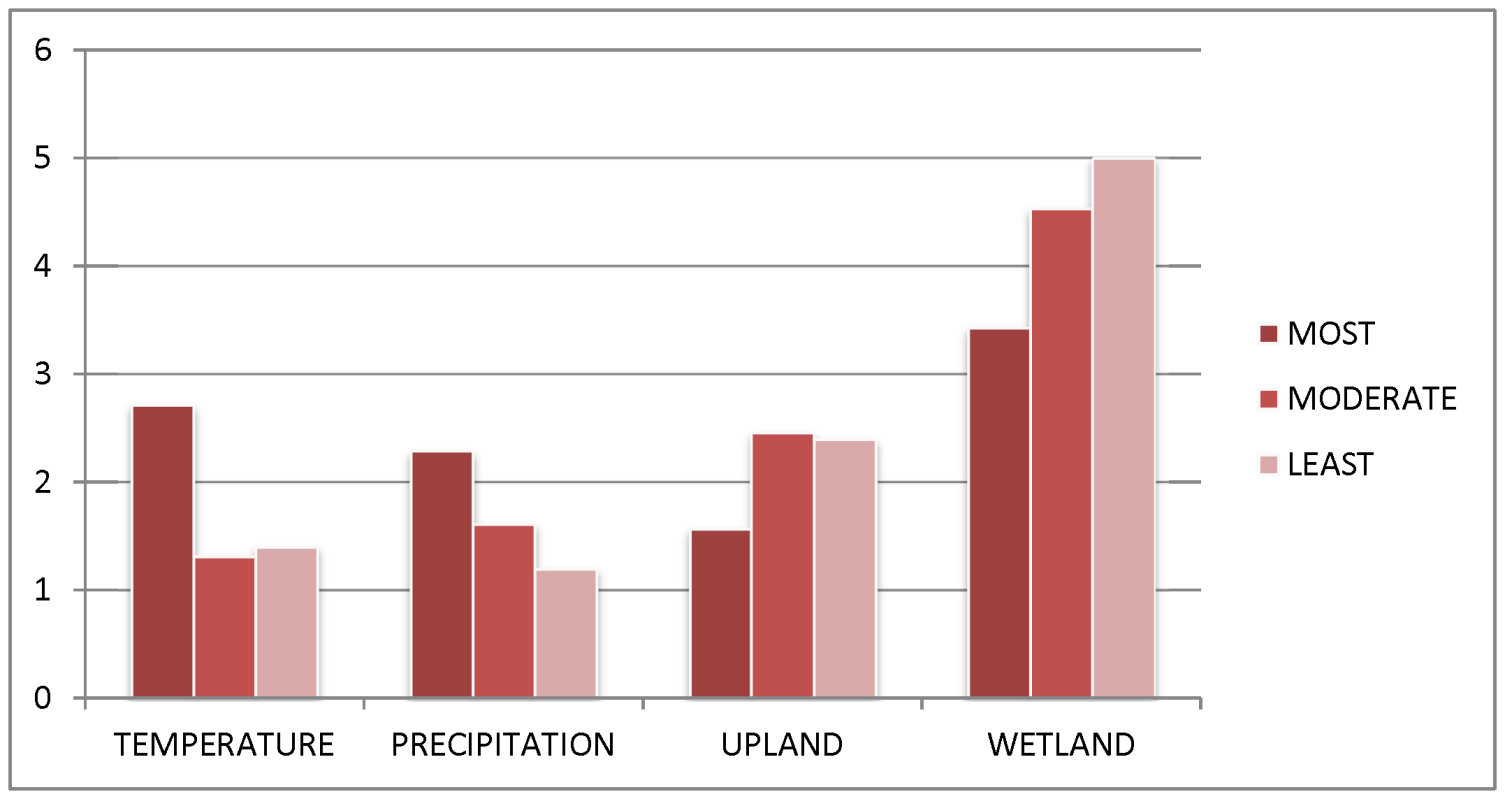
The frequency (y-axis) of variable type (x-axis) in top ten variables for waterbird species distribution models, grouped by species sensitivity to climate change. The most sensitive species were projected to lose ≥66% of their current habitat; moderately sensitive species 33–65%; and least sensitive <33%.

Temperature and precipitation predictor variables were more often in the top ten variables for the species with the greatest expected declines (Figure 5). Conversely, wetland and upland land cover variables were more often in the top ten variables for the species with smallest expected declines.

### Variable Importance: main models versus dry-years models

For dry-years models, climate predictor variables represented 45% of the top ten variables across the 22 species versus 31% for the same 22 species in the main models (Tables 4 and 5). Of land cover predictors, 65% included wetland variables in the top ten variables in the dry years and a similar 67% in the main models. However, representation of different wetland types varied with more seasonal wetlands (positive relationships only) appearing in the non-drought years (30% versus 17%) and more lakes included in the dry years (32% versus 20%).

**Table 5:** Variable importance from 22 Random Forest species distribution models for the particularly dry period, 1987–1992.

Rank	Canada Goose	Mallard	Blue-winged Teal	Northern Shoveler	Northern Pintail	Pied-billed Grebe	American Bittern	Sora	American Coot	Killdeer	Upland Sandpiper
1	P-5yr_sd(+)	W-semi(+)	W-total(+)	W-semi(+)	U-tree(-)	W-lake(+)	U-grass(+)	T-10yr_sd(+)	W-semi(+)	P-10yr_sd(-)	U-grass(+)
2	T-sum(+)	W-total(+)	W-semi(+)	T-1yr(m)	W-semi(+)	U-crop(-)	W-semi(+)	T-sum(-)	W-lake(+)	T-5yr_sd(-)	U-tree(-)
3	T-spr(-)	W-pal(+)	U-crop(-)	T-wint(m)	T-spr(-)	U-grass(+)	W-total(+)	W-river(-)	W-total(+)	U-grass(+)	W-seas(+)
4	T-10yr(-)	W-river(-)	W-lake(+)	W-total(+)	P-10yr(-)	W-semi(+)	U-crop(-)	W-total(+)	W-pal(+)	U-devel(+)	U-crop(-)
5	P-10yr_sd(-)	U-crop(-)	P-fall(+)	W-lake(+)	T-1yr(-)	W-seas(m)	W-lake(+)	P-5yr_sd(-)	U-crop(-)	T-fall(-)	P-10yr(-)
6	T-1yr(-)	P-10yr(-)	W-pal(+)	P-10yr_sd(-)	P-fall(+)	W-total(+)	W-pal(+)	W-pal(+)	T-5yr_sd(+)	W-semi(+)	W-total(+)
7	T-wint(-)	W-temp(+)	U-grass(+)	T-5yr(-)	W-total(+)	T-wint(+)	T-sum(-)	W-seas(+)	T-10yr_sd(+)	T-sum(+)	W-pal(+)
8	T-5yr(-)	T-wint(+)	T-wint(+)	U-crop(-)	T-5yr(-)	P-5yr_sd(-)	U-devel(-)	U-tree(-)	P-spr(-)	U-tree(-)	W-semi(+)
9	W-seas(-)	T-spr(∼)	P-sum(+)	T-10yr(-)	T-wint(-)	W-pal(+)	P-fall(+)	W-temp(+)	W-temp(+)	T-5yr(-)	W-temp(+)
10	P-10yr(-)	W-shrub(-)	T-sum(-)	W-seas(-)	W-pal(+)	U-devel(-)	W-seas(+)	W-lake(+)	W-seas(m)	P-1yr(-)	T-wint(+)
Rank	Willet	Marbled Godwit	Wilson's Snipe	Franklin's Gull	Ring-billed Gull	Black Tern	Sedge Wren	Marsh Wren	Common Yellow-throat	Song Sparrow	Yellow-headed Blackbird
1	P-10yr(-)	W-lake(+)	W-lake(+)	W-total(+)	W-lake(+)	U-crop(-)	T-sum(-)	W-semi(+)	W-semi(+)	U-grass(-)	W-semi(+)
2	P-5yr(-)	U-tree(-)	W-total(+)	W-lake(+)	U-crop(-)	W-pal(+)	W-total(+)	W-total(+)	U-crop(-)	U-tree(+)	P-10yr(-)
3	W-total(+)	W-total(+)	T-10yr(-)	T-10yr(-)	P-fall(+)	W-lake(-)	T-1yr(-)	W-lake(+)	W-temp(+)	W-semi(-)	W-pal(+)
4	U-tree(-)	P-10yr(-)	T-5yr(-)	P-fall(∼)	P-10yr(-)	W-temp(+)	T-10yr_sd(-)	U-grass(+)	W-pal(+)	U-crop(+)	W-total(+)
5	W-semi(+)	U-crop(-)	P-5yr_sd(-)	P-5yr_sd(+)	P-spr(-)	W-semi(+)	W-lake(+)	W-pal(+)	W-total(+)	P-sum(+)	P-5yr(-)
6	W-pal(+)	W-river(-)	W-temp(+)	T-10yr_sd(-)	U-grass(+)	T-5yr_sd(+)	T-10yr(-)	U-crop(-)	U-tree(+)	P-10yr(+)	W-seas(+)
7	W-seas(+)	T-10yr(-)	T-1yr(-)	T-spr(-)	P-5yr(-)	W-total(+)	U-crop(-)	T-10yr(+)	P-5yr_sd(-)	P-1yr(+)	U-crop(-)
8	P-10yr_sd(-)	P-5yr(-)	U-tree(-)	T-5yr(-)	T-fall(-)	T-spr(-)	P-10yr(+)	T-fall(+)	W-lake(+)	U-devel(+)	U-grass(+)
9	T-sum(m)	T-5yr(-)	W-seas(-)	T-1yr(-)	P-10yr(-)	T-fall(m)	P-5yr(+)	T-5yr(+)	T-5yr(-)	T-10yr(-)	P-10yr_sd(-)
10	U-crop(-)	W-pal(+)	T-spr(-)	U-devel(-)	P-10yr_sd(-)	T-5yr(-)	W-forest(+)	W-seas(-)	W-seas(+)	T-5yr(-)	U-tree(-)

Top ten variables shown in descending order of rank. Variable categories were denoted by W (wetland), U (upland), P (precipitation), and T (temperature). Signs indicated the relationship between the predictor and the species response: + (positive), - (negative), m (unimodel), and ∼ (equivocal).

## Discussion

Our projections of large range reductions for waterbirds breeding in the Prairie Pothole Region are not surprising. Globally, freshwater habitats are expected to be particularly vulnerable to climate change [36]. If, as the future climate projections we used indicate, temperatures rise by ∼3.0°C and precipitation rises only by 3% by mid-century in the Prairie Pothole Region, many fewer pothole wetlands will exist on the landscape due to an increased deficit in precipitation relative to evapotranspiration. Similarly, other studies of the Prairie Pothole Region have projected a drier future and concomitant reductions in waterbird habitat [5], [8], [37].

Past studies in the Prairie Pothole Region that extrapolated from relations between climatic factors and wetlands inferred generalized habitat losses for waterfowl [5], [8]. Our species-specific approach indicated large variability in the vulnerabilities of waterbird species to climate change. This is expected as patterns of waterbird habitat selection vary among species for wetland attributes such as size, permanence, and vegetative cover [10], [38]. Hydrological studies indicate that temperature and precipitation regimes affect not only the number of wetlands and wetland size, but marsh vegetation dynamics and the vegetative coverage patterns at the landscape scale [5]. While reducing the overall number of wetlands, a drier climate will likely lead to more extensive coverage of wetlands by dense vegetation rather than wetland conditions characterized by a mixture of open water and vegetation [5]. Species are expected to respond differentially to these changes in wetland characteristics. Furthermore, individualistic species' responses appear the norm [39], [40], [41].

Our projections of future change were not always consistent with documented waterbird responses to a historic dry period which represented one possible expression of a drier climate. The dry historic period, a consequence of reduced precipitation, is not a direct analog of future drying which is expected to be driven by increases in evapotranspiration (Figure 2) [42]. Thus, it is unclear to what extent the historic pattern of drought can be used as a benchmark for future climate change. Therefore, the inconsistencies between the dry historic waterbird response relative to projected future responses may indicate our models are under- or over- estimating waterbird response to climate change for some species. It is also possible that changes in temperature versus precipitation may result in divergent, and unprecedented, future wetland habitat conditions. In that case, divergent waterbird responses, relative to the past responses, would not be surprising.

The historic range of temperature variability did not overlap future projections and so, our SDMs were projecting beyond known climatic boundary conditions. Model extrapolation to novel conditions is common when projecting species response to future climate [43]. Our single values for yearly averages (Figure 2), indicated almost no overlap in temperature range between historic and projected time intervals. However, because of spatial variation in temperature regimes (i.e., warmer in the south, as shown in Figure 3), there were likely many individual grid cells in which future temperatures overlapped the historic range even if the study area yearly means do not. SDMs based on the Random Forest algorithm are constrained when extrapolating beyond the observed values of the predictor variables. For example, when projected temperatures are outside of the range of the training set the algorithm holds the prediction constant at the last known value of temperature [44]. Therefore, if future wetland habitat conditions selected by the species become less common with increased temperatures, our estimates of habitat losses for many species may be underestimates.

Ranking predictor variables by their importance provides additional insights into how the 31 waterbird species may respond to changing environmental factors. We included predictors related to suitable waterbird habitat quality, including the amount and type of wetland basins, and temporally scaled temperature and precipitation covariates. Species projected to be most sensitive to anticipated climate change (changes in temperature and precipitation,Table 4) consistently reflected the ecology of the species. For example, the two diving ducks, Ruddy Duck and Redhead, primarily selected large wetlands, such as semipermanent basins, and were less susceptible to total drying [5], [45]. As a consequence of their habitat associations, no climate covariates ranked in the top ten for these two waterbird species. In contrast, waterbirds that rely on shallow water habitat, such as Sora or Sedge Wren, or dynamic habitat such as Black Tern or Mallard, showed a much greater projected change in distribution to future climatic conditions [5], [38].

The variable importance ranks also suggested that waterbirds may shift their habitat preferences with increased drying. More climate covariates and more permanent wetland regimes appeared in the top variables for dry years. In the Prairie Pothole Region, wetland function can rapidly change with significant changes in the climate. In dry years, for example, semipermanent wetlands may function more like seasonal wetlands, and seasonal wetlands more like temporary wetlands. This differential sensitivity to climate change explains why seasonal wetlands were less important and lakes more important in dry years.

Because bioclimatic SDMs are generally exploratory with many collinear climate predictors, there is concern that these models over-fit the data and thus misrepresent species distributions [46]. However, the inclusion of many collinear climate predictors is often warranted when causal links between specific climate predictors and species' distributions are not established, leading to better model fit and projections [47]. We found that when we reduced our 18 climate and 13 land cover variables to 14 uncorrelated climate and 10 uncorrelated land cover variables model projections were similar: 45% average range reduction for the full model and 48% for the reduced model (results not shown).

## Conclusions

Our results indicated, on average, large decreases in suitable habitat by the 2040s for 31 waterbird species breeding in the Prairie Pothole Region of the U.S.A. Importantly, our results were consistent between two contrasting future climate scenarios. However, there was substantial variability in species specific responses to projected climate change. Therefore, strategic efforts to mitigate climate change effects should preferentially direct management actions to those species expected to be most vulnerable. In continuing research, we are exploring in greater detail various sources of uncertainty in our projections including additional model algorithms, alternative covariates, and other sources of species distribution data [48].

## Supporting Information

Figure S1 **Distribution of grassland and palustrine wetlands on the U.S. Prairie Pothole Region landscape.** Darker shades represent greater coverage of grassland (versus cropland) and greater areal coverage of wetlands (log transformed).
(TIF)

Figure S2 **Map of species distributions for baseline and two future climate projections.** Brown indicates areas where the species is predicted to occur and green represents areas where the species is not predicted to occur.
(TIFF)

Figure S3 **Map of species distributions for baseline and two future climate projections.** Brown indicates areas where the species is predicted to occur and green represents areas where the species is not predicted to occur.
(TIF)

Figure S4 **Map of species distributions for baseline and two future climate projections.** Brown indicates areas where the species is predicted to occur and green represents areas where the species is not predicted to occur.
(TIF)

Figure S5 **Map of species distributions for baseline and two future climate projections**. Brown indicates areas where the species is predicted to occur and green represents areas where the species is not predicted to occur.
(TIF)

Figure S6 **Map of species distributions for baseline and two future climate projections.** Brown indicates areas where the species is predicted to occur and green represents areas where the species is not predicted to occur.
(TIF)

Figure S7 **Map of species distributions for baseline and two future climate projections.** Brown indicates areas where the species is predicted to occur and green represents areas where the species is not predicted to occur.
(TIF)

Figure S8 **Map of species distributions for baseline and two future climate projections**. Brown indicates areas where the species is predicted to occur and green represents areas where the species is not predicted to occur.
(TIF)

Figure S9 **Map of species distributions for baseline and two future climate projections**. Brown indicates areas where the species is predicted to occur and green represents areas where the species is not predicted to occur.
(TIF)

Figure S10 **Map of species distributions for baseline and two future climate projections**. Brown indicates areas where the species is predicted to occur and green represents areas where the species is not predicted to occur.
(TIF)

Figure S11 **Map of species distributions for baseline and two future climate projections.** Brown indicates areas where the species is predicted to occur and green represents areas where the species is not predicted to occur.
(TIF)

Figure S12 **Map of species distributions for baseline and two future climate projections.** Brown indicates areas where the species is predicted to occur and green represents areas where the species is not predicted to occur.
(TIF)
