# Supplementary figures and images for: Vulnerability of Breeding Waterbirds to Climate Change in the Prairie Pothole Region, U.S.A
Source: PLoS One. 2014 Jun 13;9(6):e96747. doi: 10.1371/journal.pone.0096747 (PMC4057080; doi:10.1371/journal.pone.0096747)

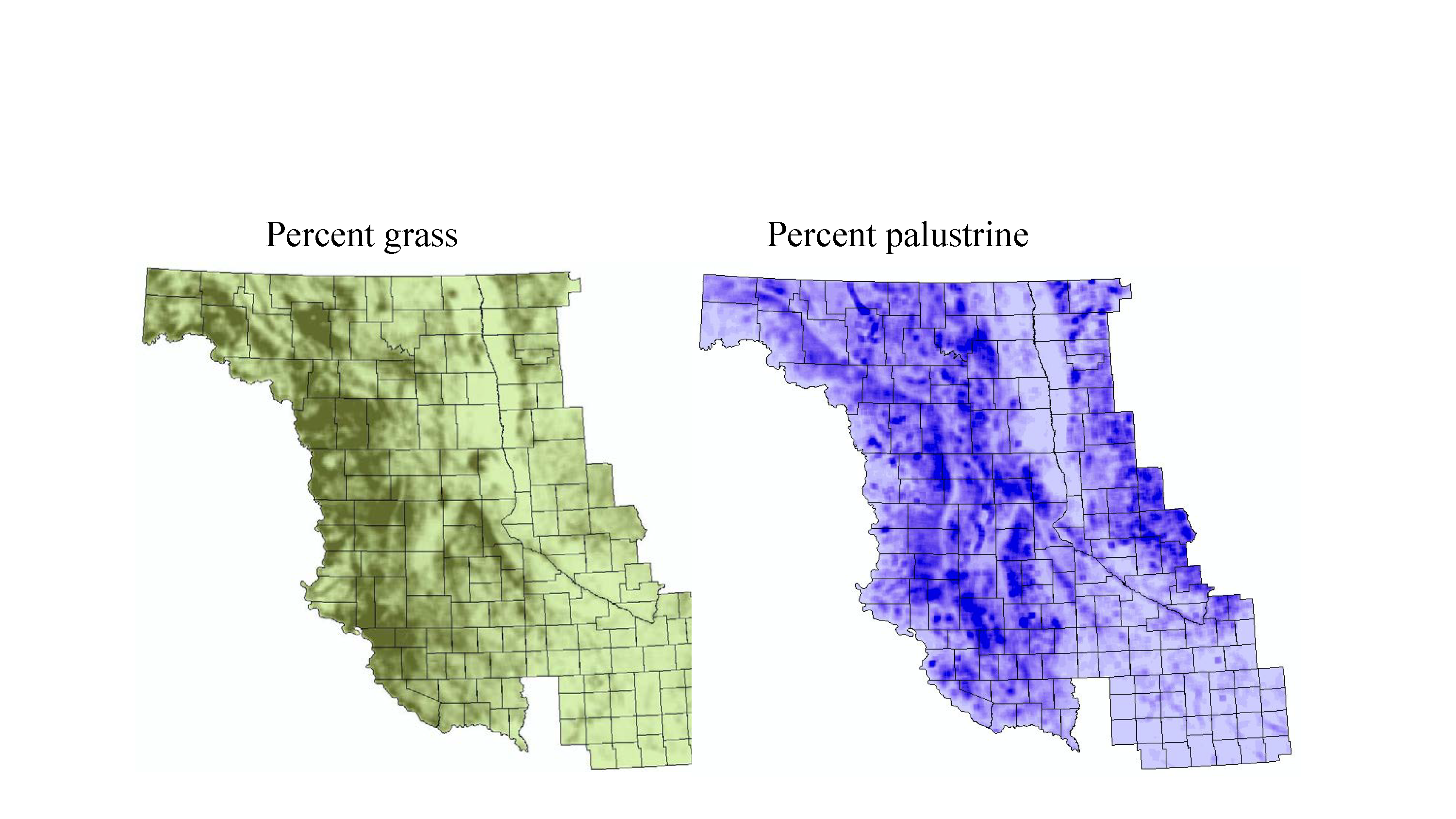

Supplement: Figure S1 — Distribution of grassland and palustrine wetlands on the U.S. Prairie Pothole Region landscape. Darker shades represent greater coverage of grassland (versus cropland) and greater areal coverage of wetlands (log transformed). (TIF) [file pone.0096747.s001.tif]

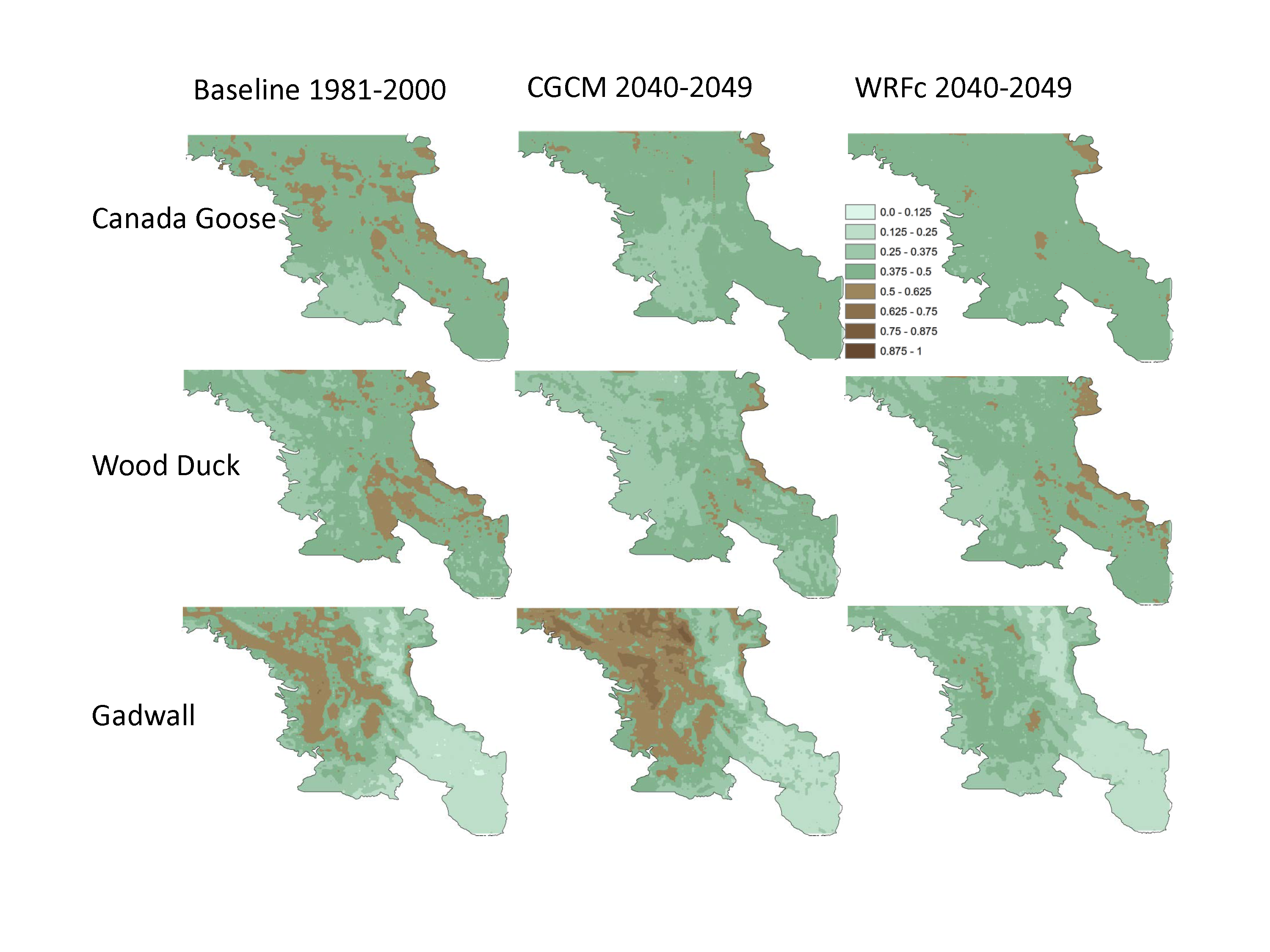

Supplement: Figure S2 — Map of species distributions for baseline and two future climate projections. Brown indicates areas where the species is predicted to occur and green represents areas where the species is not predicted to occur. (TIFF) [file pone.0096747.s002.tif]

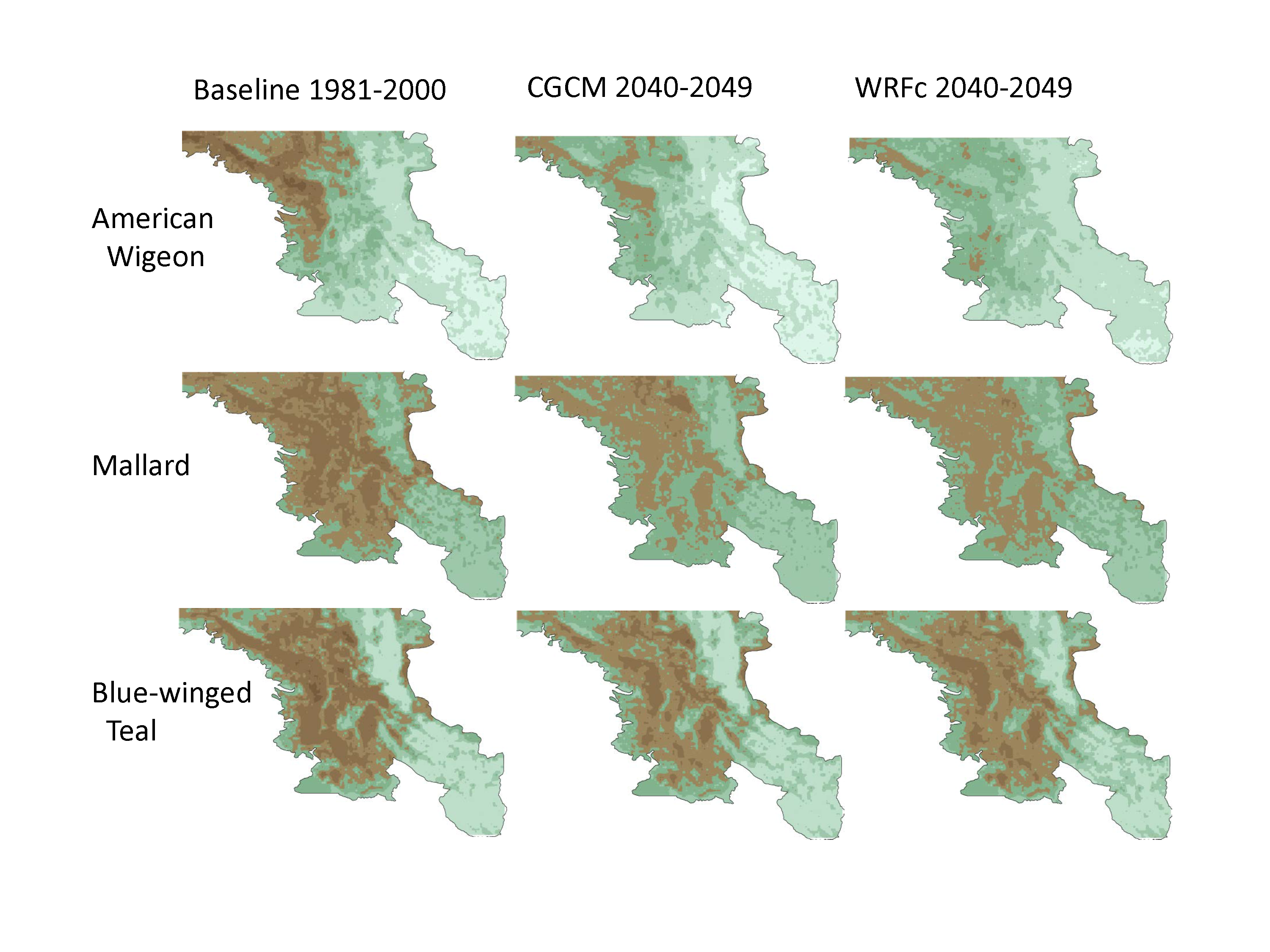

Supplement: Figure S3 — Map of species distributions for baseline and two future climate projections. Brown indicates areas where the species is predicted to occur and green represents areas where the species is not predicted to occur. (TIF) [file pone.0096747.s003.tif]

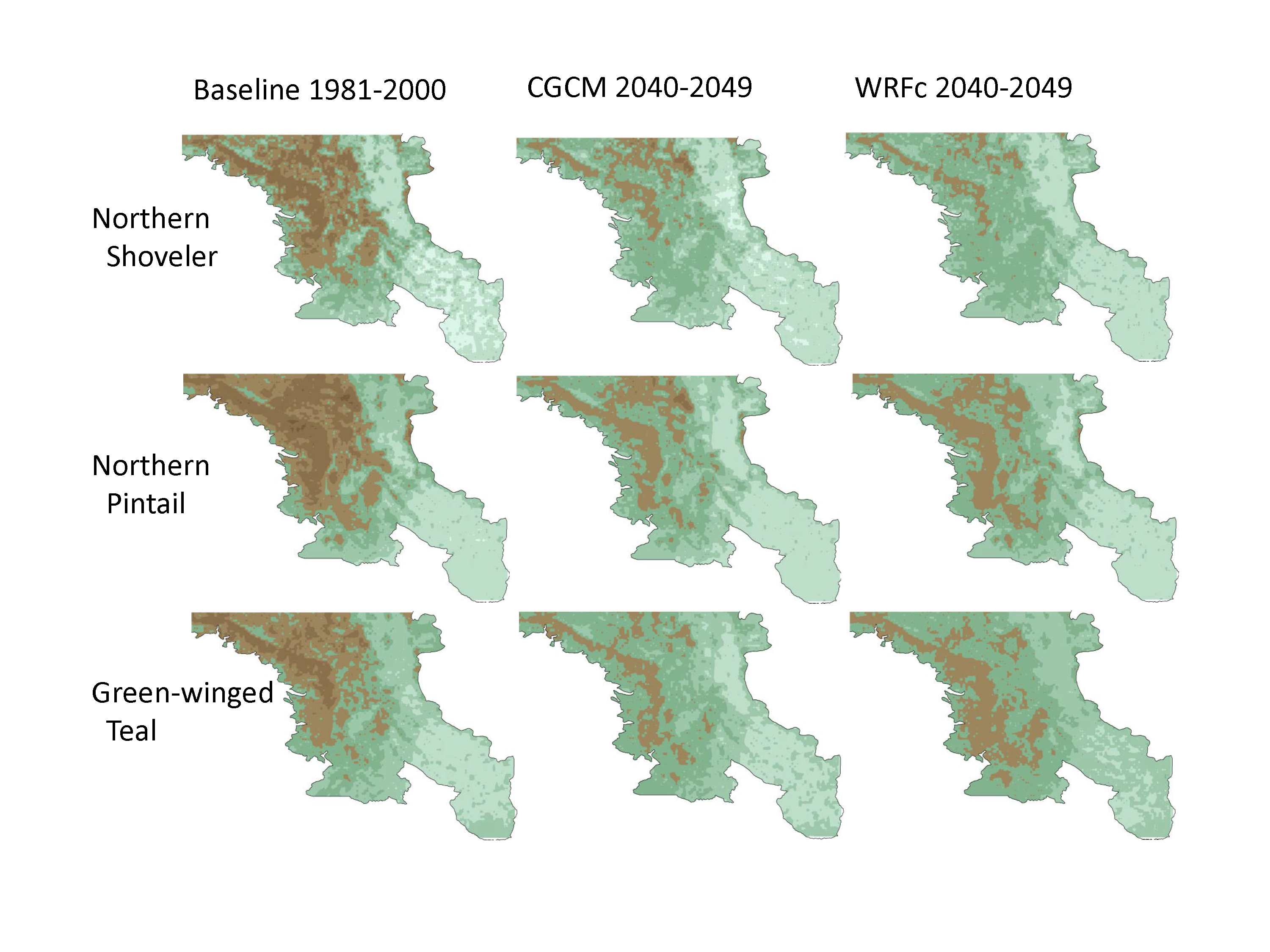

Supplement: Figure S4 — Map of species distributions for baseline and two future climate projections. Brown indicates areas where the species is predicted to occur and green represents areas where the species is not predicted to occur. (TIF) [file pone.0096747.s004.tif]

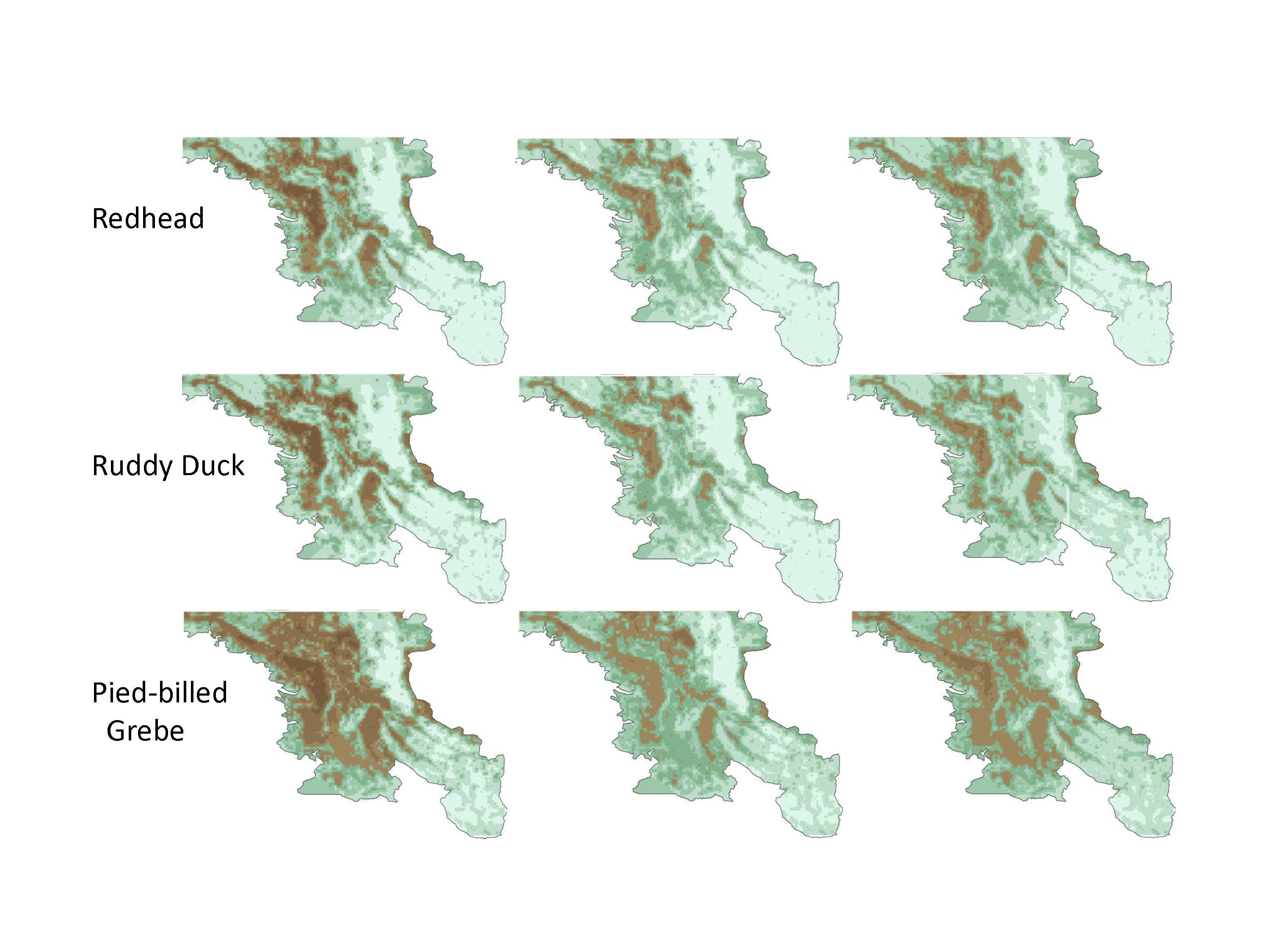

Supplement: Figure S5 — Map of species distributions for baseline and two future climate projections. Brown indicates areas where the species is predicted to occur and green represents areas where the species is not predicted to occur. (TIF) [file pone.0096747.s005.tif]

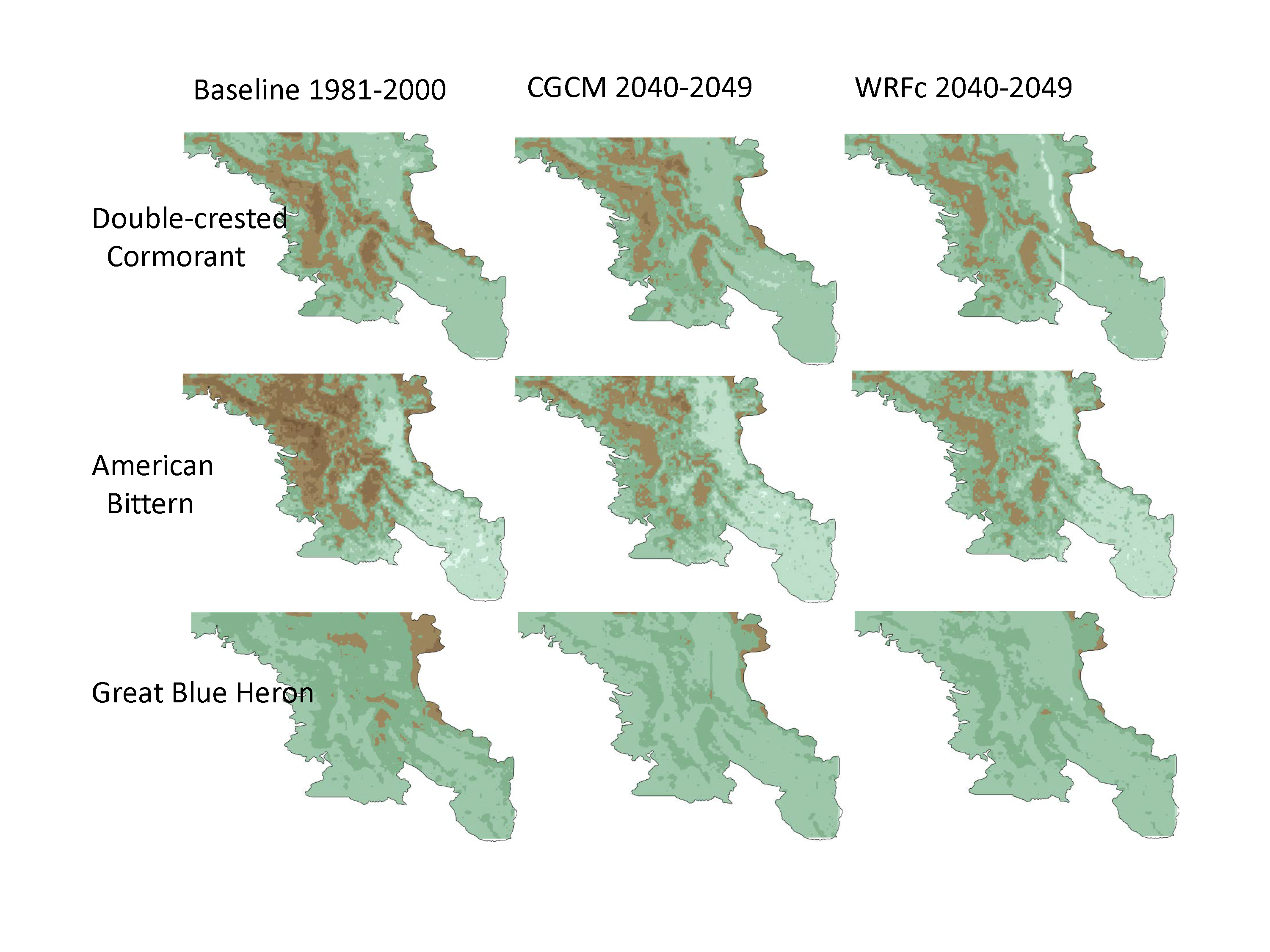

Supplement: Figure S6 — Map of species distributions for baseline and two future climate projections. Brown indicates areas where the species is predicted to occur and green represents areas where the species is not predicted to occur. (TIF) [file pone.0096747.s006.tif]

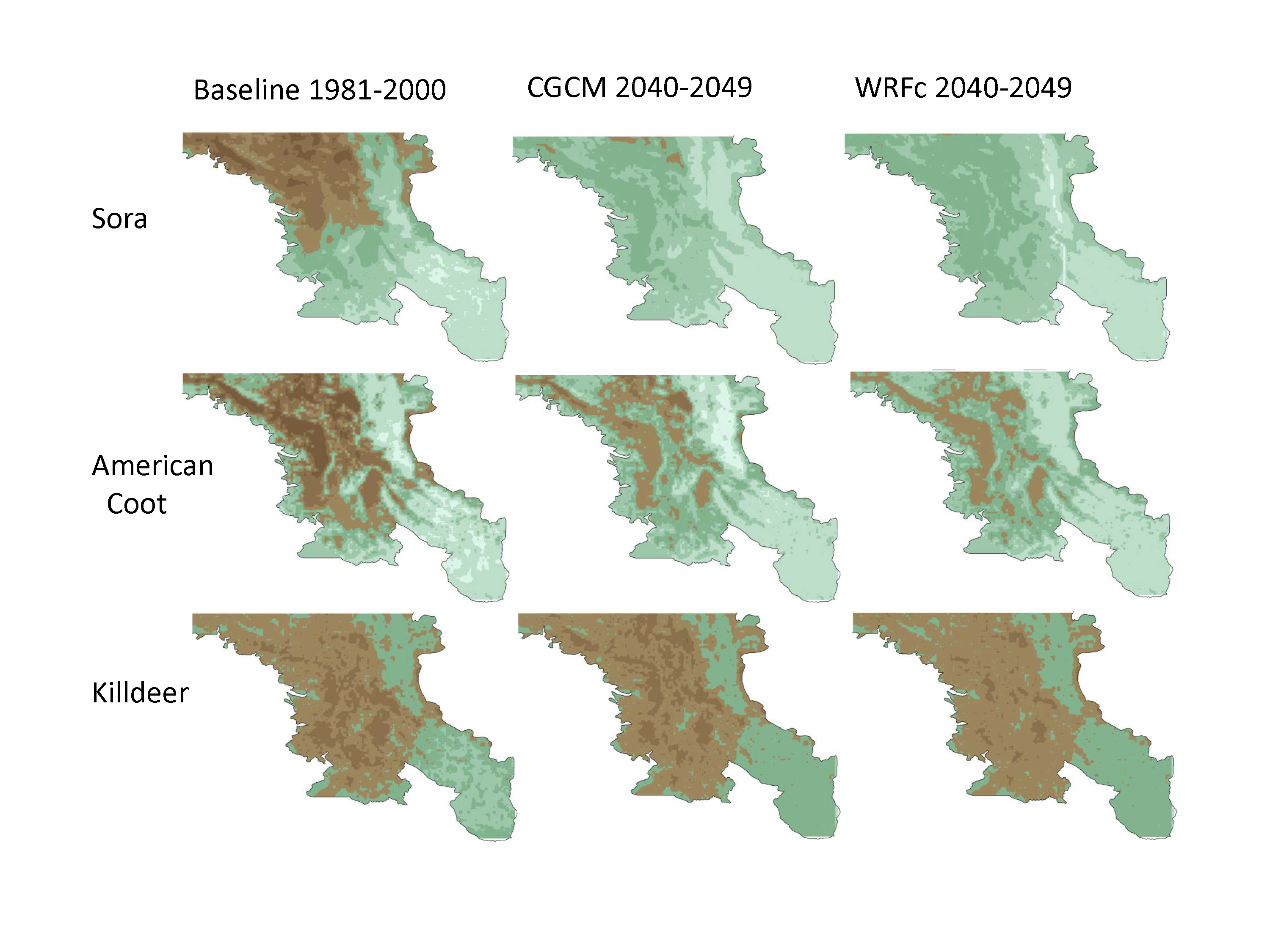

Supplement: Figure S7 — Map of species distributions for baseline and two future climate projections. Brown indicates areas where the species is predicted to occur and green represents areas where the species is not predicted to occur. (TIF) [file pone.0096747.s007.tif]

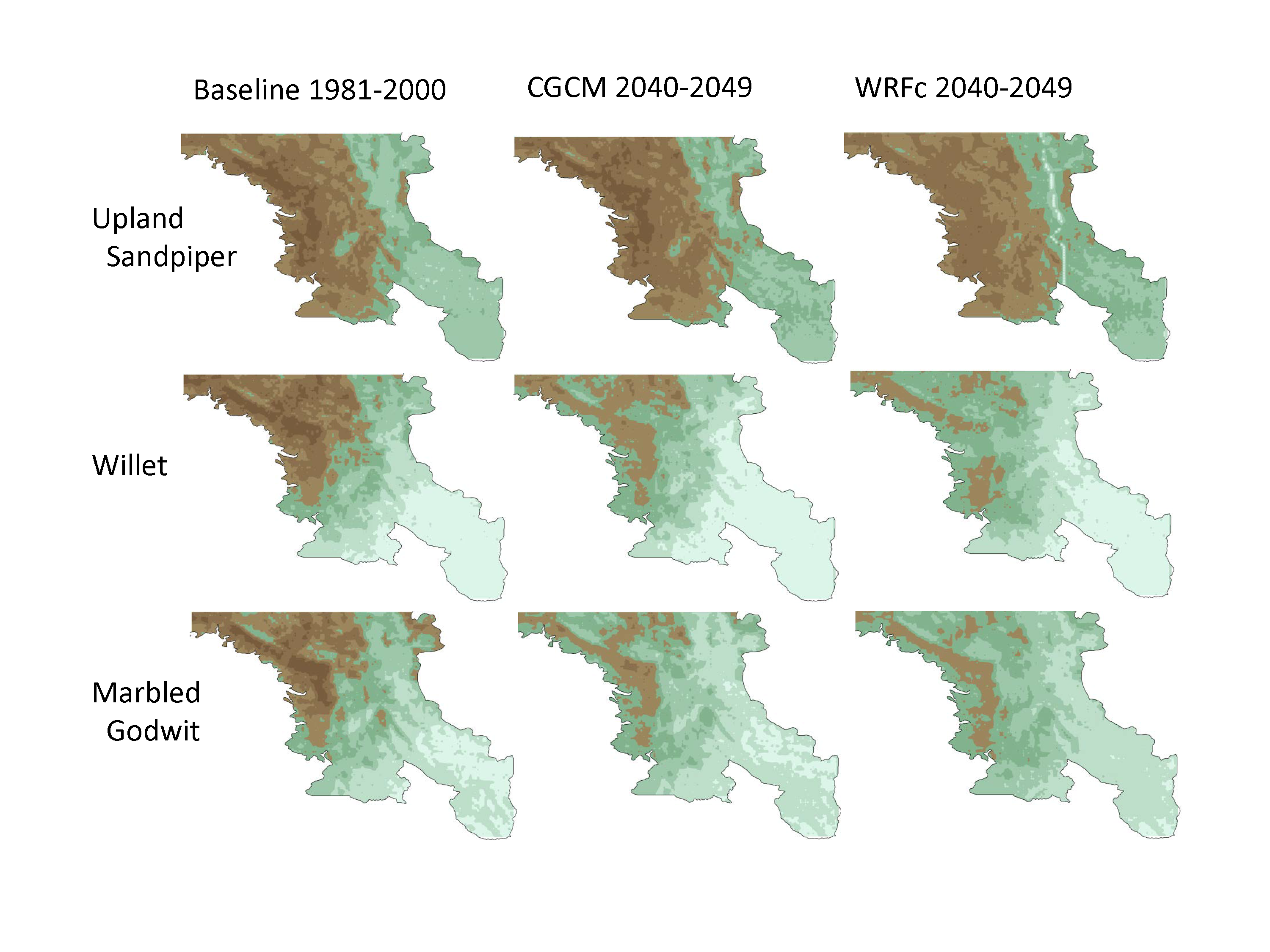

Supplement: Figure S8 — Map of species distributions for baseline and two future climate projections. Brown indicates areas where the species is predicted to occur and green represents areas where the species is not predicted to occur. (TIF) [file pone.0096747.s008.tif]

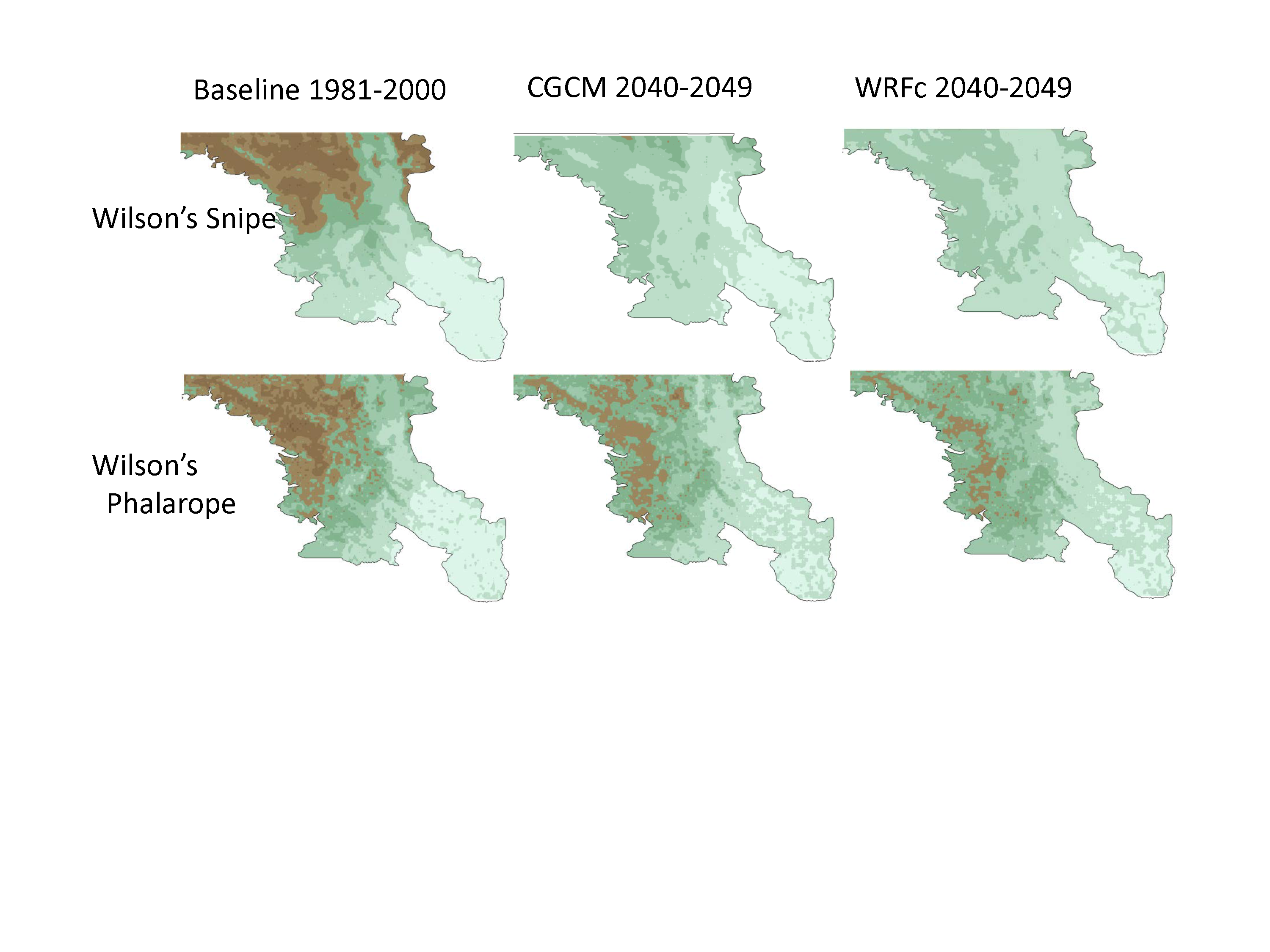

Supplement: Figure S9 — Map of species distributions for baseline and two future climate projections. Brown indicates areas where the species is predicted to occur and green represents areas where the species is not predicted to occur. (TIF) [file pone.0096747.s009.tif]

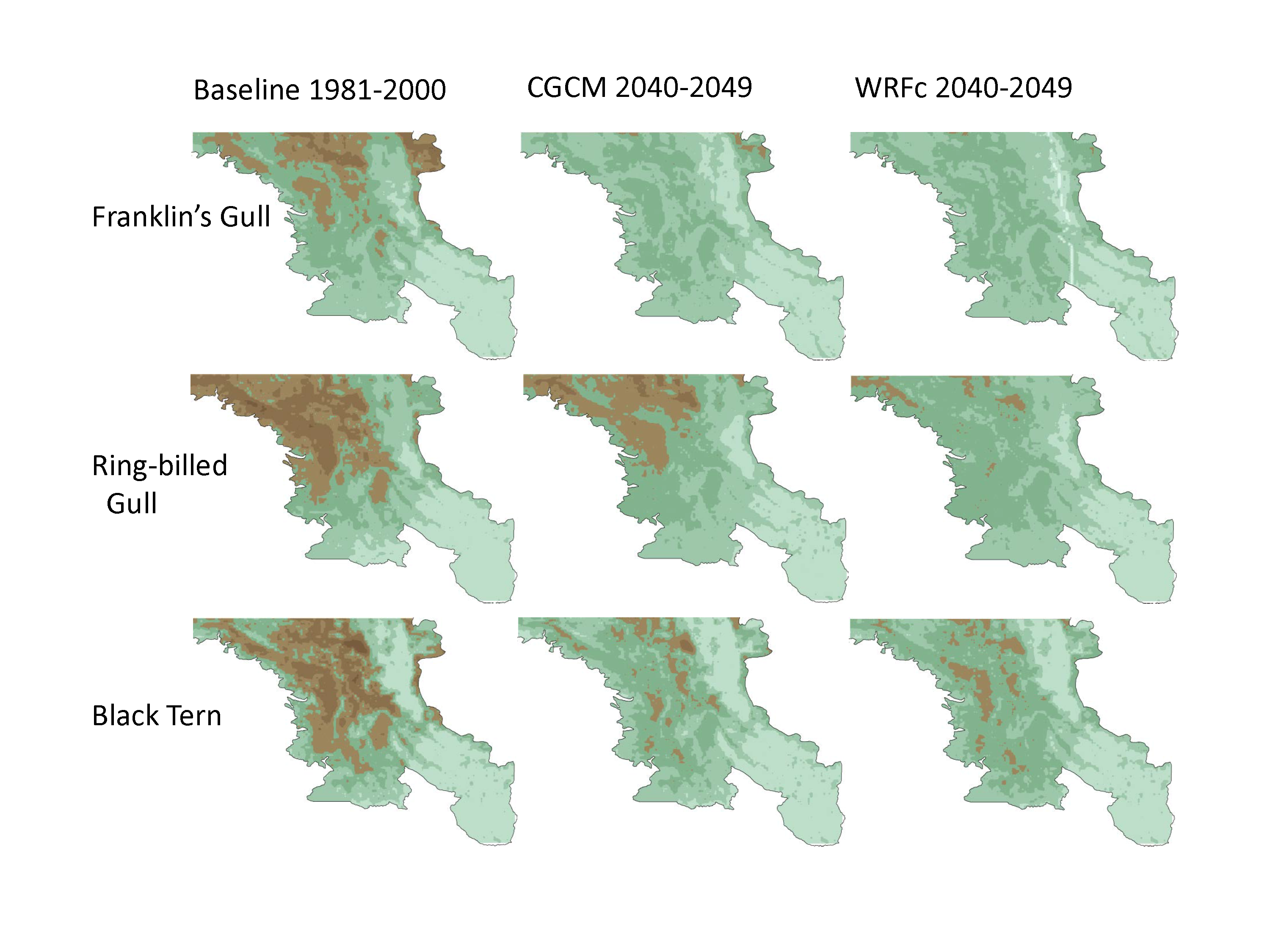

Supplement: Figure S10 — Map of species distributions for baseline and two future climate projections. Brown indicates areas where the species is predicted to occur and green represents areas where the species is not predicted to occur. (TIF) [file pone.0096747.s010.tif]

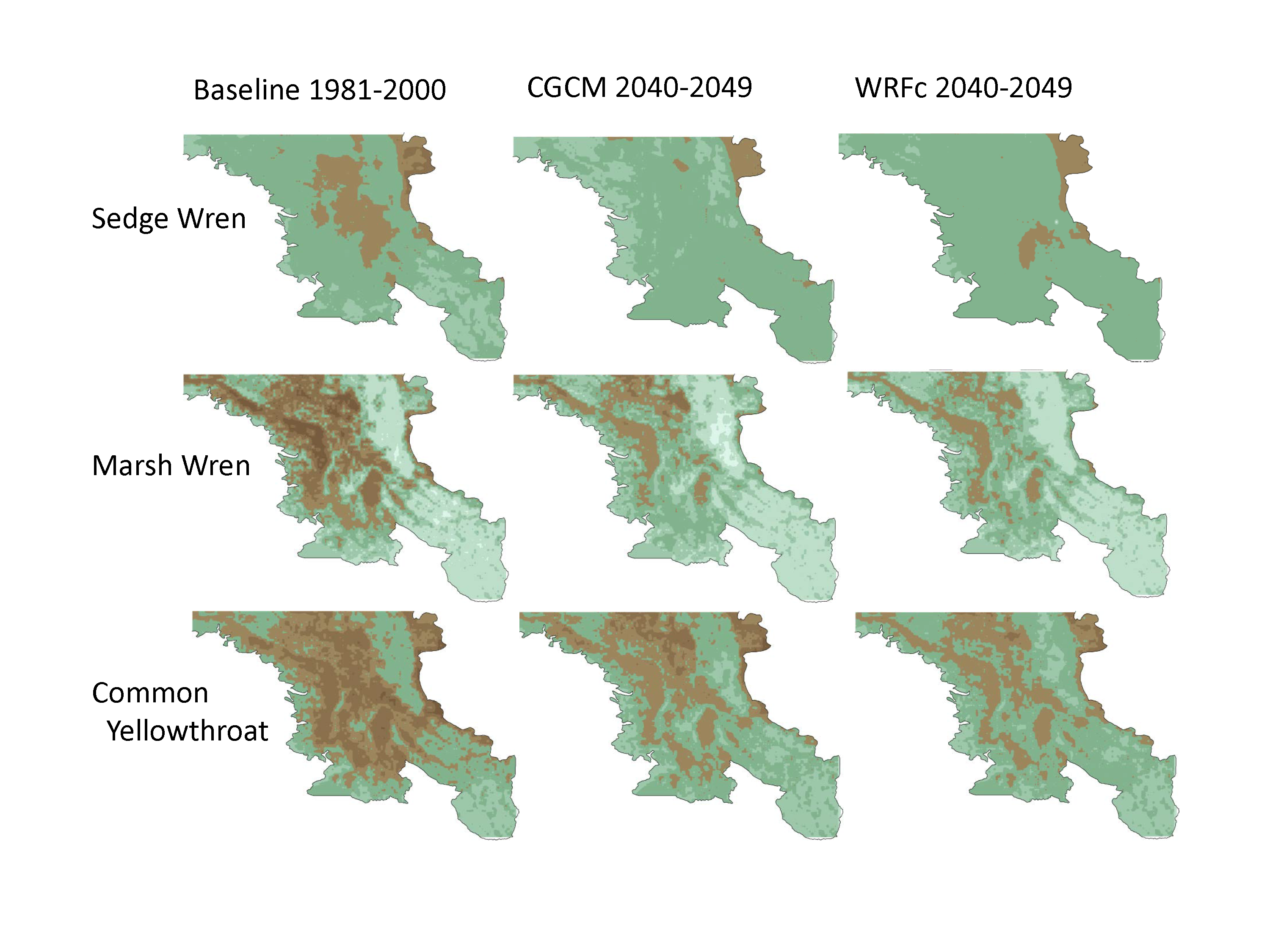

Supplement: Figure S11 — Map of species distributions for baseline and two future climate projections. Brown indicates areas where the species is predicted to occur and green represents areas where the species is not predicted to occur. (TIF) [file pone.0096747.s011.tif]

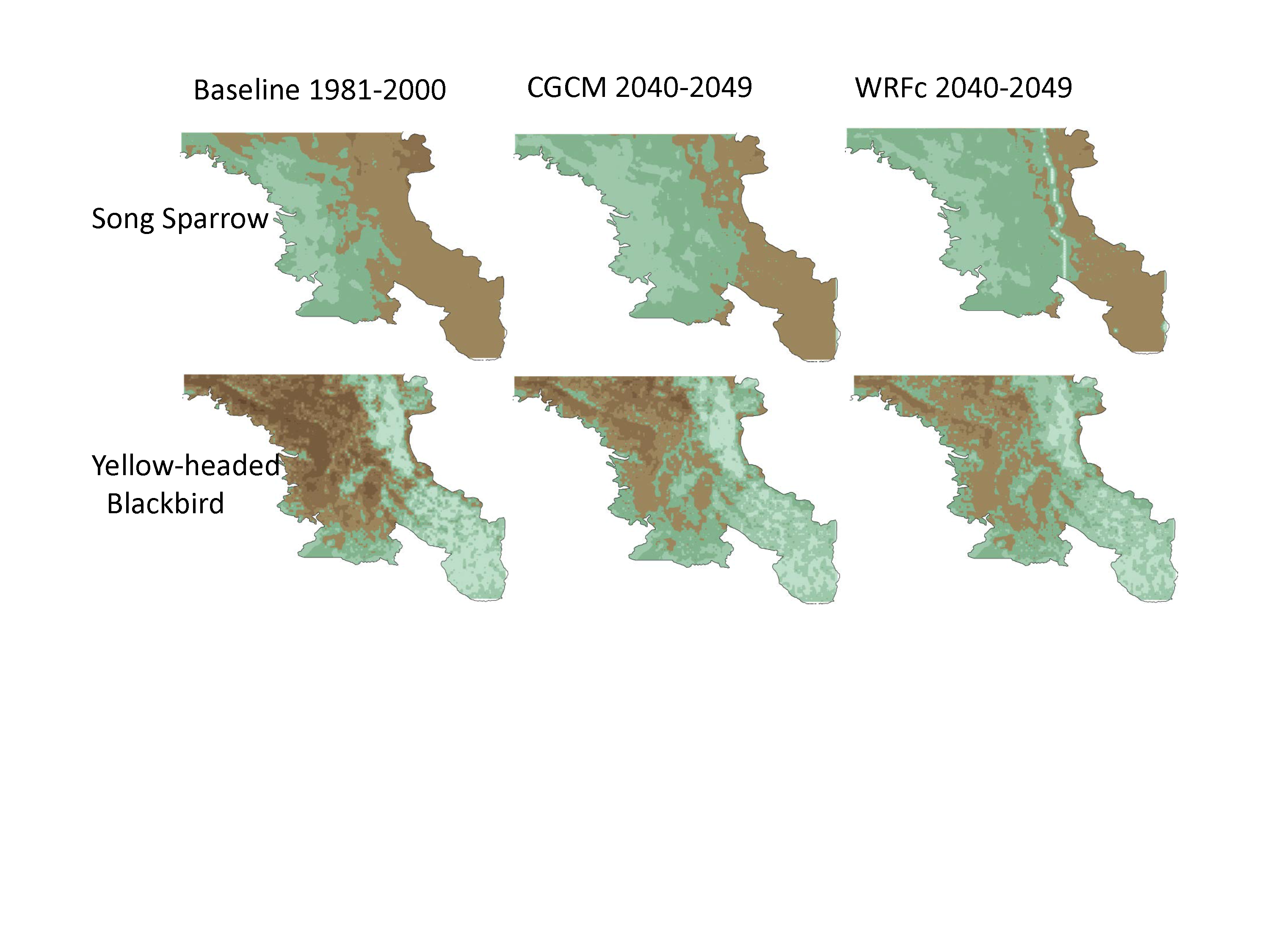

Supplement: Figure S12 — Map of species distributions for baseline and two future climate projections. Brown indicates areas where the species is predicted to occur and green represents areas where the species is not predicted to occur. (TIF) [file pone.0096747.s012.tif]
